# *DFT2FEFFIT*: a density-functional-theory-based structural toolkit to analyze EXAFS spectra

**DOI:** 10.1107/S1600576724005454

**Published:** 2024-07-17

**Authors:** Alain Manceau, Romain Brossier, Olivier Mathon, Kirill A. Lomachenko, Marius Retegan, Pieter Glatzel, Stephan N. Steinmann

**Affiliations:** a European Synchrotron Radiation Facility (ESRF) 38000Grenoble France; b ENS de Lyon, CNRS, Laboratoire de Chimie 69342Lyon France; c Université Grenoble Alpes, CNRS, ISTerre 38000Grenoble France; Australian Synchrotron, ANSTO, Australia

**Keywords:** *FEFF* software, density functional theory, DFT, apatite, rare earth elements, cerium, EXAFS

## Abstract

This article presents a Python-based program, *DFT2FEFFIT*, to regress theoretical extended X-ray absorption fine structure (EXAFS) spectra calculated from density functional theory structure models against experimental EXAFS spectra. To showcase its application, Ce-doped fluorapatite [Ca_10_(PO_4_)_6_F_2_] is revisited as a representative of a material difficult to analyze by conventional multi-shell least-squares fitting of EXAFS spectra. The software is open source and publicly available.

## Abstract 2

This paper presents *DFT2FEFFIT*, an open-source Python-based program that regresses theoretical EXAFS spectra calculated from density functional theory structure models against experimental EXAFS spectra. *DFT2FEFFIT* could be of use for researchers involved in various fields of solid-state chemistry, physics and mineralogy.

## 1. Introduction

Extended X-ray absorption fine structure (EXAFS) spectroscopy is an established method for characterization of the local structure of liquids, glasses and crystalline materials (Chantler *et al.*, 2020 ▸). The chemical nature, number and distance of atoms located in successive spherical shells around the X-ray photoabsorber are obtained by fitting the experimental EXAFS signal to the theoretical χ(*k*) function (Stern *et al.*, 1975 ▸; Rehr & Albers, 2000 ▸; Rehr *et al.*, 2020 ▸):
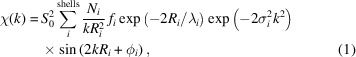
where *k* is the photoelectron wavenumber, *k* = 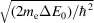
*m*_e_ is the mass of the electron, Δ*E*_0_ is the shift in the Fermi level between experiment and theory, 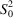 is a scale factor taking into account amplitude damping due to multielectron effects, the sum is over shells of atoms of a particular type *i* and at similar distance from the photoabsorber, *N_i_* is the coordination number, *R_i_* is the interatomic distance, *f_i_* is the photoelectron backscattered amplitude, λ*_i_* is the mean free path of the photoelectron, 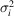 is the mean-square radial displacement of atoms in the *i*th shell (Debye–Waller term), and ϕ*_i_* is the phase shift of the electronic wave. Although equation (1), strictly speaking, applies only to single scattering paths from neighboring shells of atoms, Rehr & Albers (1990 ▸) showed that this formula can be generalized to represent the contribution from *N* equivalent multiple scattering contributions of path length 2*R*.

Characterizing the local structure requires solving the inverse problem of finding a plausible structure model that corresponds to the measured EXAFS signal (Timoshenko *et al.*, 2019 ▸; Terry *et al.*, 2021 ▸). As powerful a structural method as EXAFS is, the analysis of chemically complex and structurally defective materials is challenging (Boyanov *et al.*, 1996 ▸). Because the information content of quality EXAFS data is typically bandwidth-limited to about *k*_max_ ≃ 14 Å^−1^, two overlapping subshells separated by less than ∼0.10–0.15 Å are unresolved in multi-elemental materials. Furthermore, EXAFS fails to distinguish neighboring atoms of similar scattering power and phase shifts (Δ*Z* < ∼10). Yet another difficulty arises when the interatomic distances in an atomic shell are unequal. In equation (1), the radial distribution function (RDF) of the atoms in shell *i* is assumed to be Gaussian,
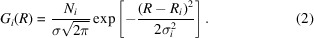


Poorly crystalline and compositionally heterogeneous materials frequently have more complicated analytical atomic distributions than Gaussian. An asymmetric distribution of distances results in an apparent loss of coordination and usually reinforces correlations between the *N* and σ parameters in the fit (Marcus *et al.*, 1986 ▸; Crozier, 1997 ▸). Still, the asymmetric shape of the distribution may be obtained by a cumulant EXAFS analysis of the disordered shell, but this model-independent method is limited to small degrees of disorder when the cumulant series rapidly converges within the EXAFS *k* range (Dalba & Fornasini, 1997 ▸).

**Figure 1:**
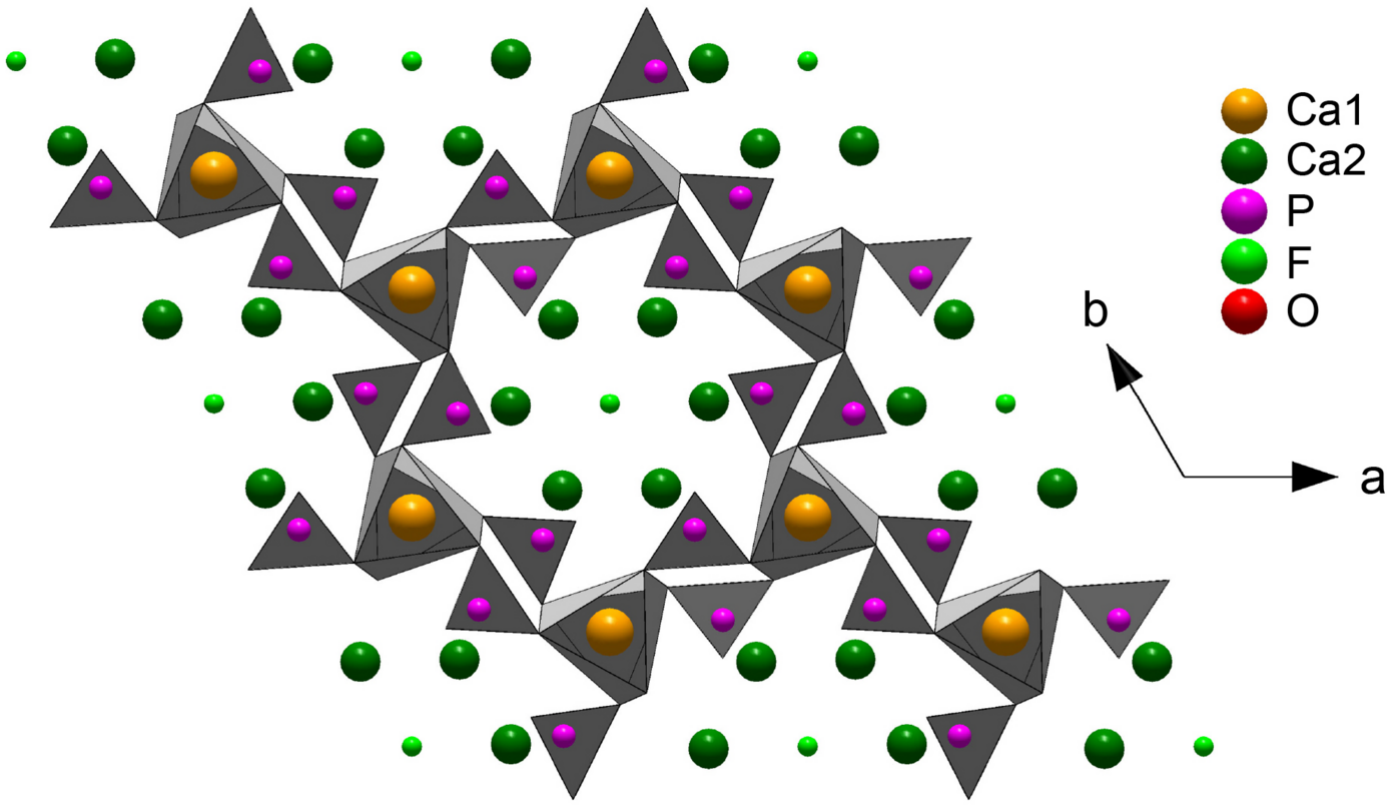
The structure of FAp projected in the *ab* plane (Hughes *et al.*, 1989 ▸; Harlov & Rakovan, 2015 ▸). The F atom is located in the middle of the tunnel.

**Figure 2:**
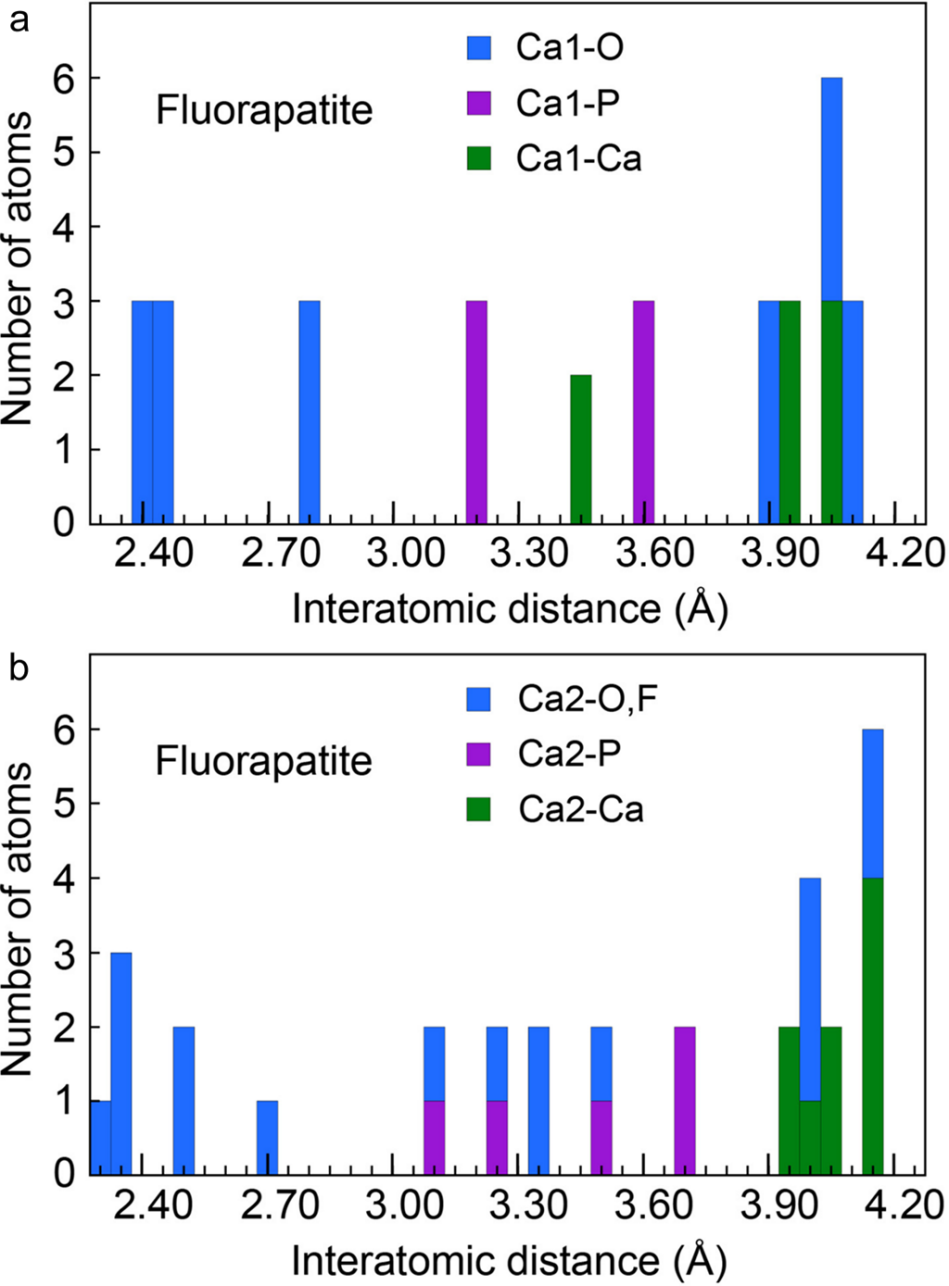
Population histograms of (*a*) the Ca1—(O,P,Ca) and (*b*) the Ca2—(O,F,P,Ca) distances in fluorapatite (Hughes *et al.*, 1989 ▸). The number of atoms is counted in intervals of 0.05 Å.

A prototypical case of a material difficult to analyze by EXAFS is fluorapatite [Ca_10_(PO4)_6_F_2_, FAp]. Its structure comprises two Ca sites: a larger nine-coordinated Ca1 site forming with the phosphate groups the walls of a honeycomb framework, and a smaller seven-coordinated Ca2 site along the sub-nanometre-sized tunnels containing the column F site (Hughes *et al.*, 1989 ▸) (Fig. 1 ▸). The coordination of Ca1 is really 6 + 3 rather than 9, and that of Ca2 is 6 + 1, and the six Ca1—O and six Ca2—(O,F) distances are unequal, which is a source of uncertainty in the determination of the site occupancy of a substituent (Fig. 2 ▸). The situation is not improved beyond the first coordination shell, because the Ca—O, Ca—P and Ca—Ca distances are widely distributed and partly overlap.

Natural FAp is commonly enriched in trivalent rare earth elements (REE) (Harlov & Rakovan, 2015 ▸; Manceau *et al.*, 2022 ▸). The substitution may occur on the Ca1 or Ca2 site, depending on the ionization energy of the substituent (Manceau *et al.*, 2024 ▸). The charge excess resulting from REE^3+^ for Ca^2+^ substitution is generally considered to be balanced by parallel Na^+^ ↔ Ca^2+^ substitution on the Ca1 or Ca2 octahedral site, or Si^4+^ ↔ P^5+^ substitution on the tetrahedral site (Rønsbo, 1989 ▸; Fleet *et al.*, 2000 ▸). Furthermore, the charge balance may occur locally, or indifferently at a short- or long-range distance. Other substitutional mechanisms can be envisaged, such as a coupled REE^3+^ + F^−^ ↔ Ca^2+^ substitution with incorporation of an additional F^−^ ion in the FAp tunnels, and a coupled 2REE^3+^ + Vac → 3Ca^2+^ substitution with creation of a Ca vacancy. Clearly, the conventional multi-shell EXAFS fitting approach has a high risk of failing to find the correct local structure of REE due to the inherent large number of unknowns to fit with multiple optima in parameter space. Not all of the atomic shells can be refined independently without causing correlations between parameters. Hence, *a priori* information is required to make educated guesses. Another inherent problem, besides the non-uniqueness of the model parameters resulting from overlapping subshells, is the lack of discrimination between Si and P backscatterers, and the low sensitivity of EXAFS spectroscopy to F, Na and vacancies.

An alternative to multi-shell EXAFS fitting is to use the geometric constraints of density functional theory (DFT) models for comparative modeling of the EXAFS spectra (Harris *et al.*, 2006 ▸; Cotelesage *et al.*, 2012 ▸). The EXAFS signal is a one-dimensional projection in reciprocal space of a spherically averaged three-dimensional structure. Incorporation of an impurity in a solid modifies not just its atomic pair distances but also those of its neighboring atoms and its bond angles. This information is compressed in EXAFS data and not easily and reliably accessible, motivating the use of DFT models as three-dimensional templates of the whole impurity environment. Recently, we followed this approach and showed by calculating the EXAFS spectra of DFT models that Ce^3+^ occupies the Ca2 site in FAp with a coupled Si^4+^ substituent at a short distance [*d*(Ce2—Si) = 3.09 Å, Ce2–Si-close model], while the coupled Na^+^ ↔ Ca^2+^ substitution on the Ca1 or Ca2 octahedral site was negated (Manceau *et al.*, 2024 ▸).

Here, we extend our previous approach and present *DFT2FEFFIT*, a general regression analysis tool that best-fits an EXAFS spectrum using the χ*_i_* functions generated by *FEFF* (Version 8.2; Ankudinov & Rehr, 1997 ▸) from a DFT model. Its capabilities are demonstrated with reconstructions of the Ce *L*_3_ edge EXAFS spectrum of the FAp reference from Cerro de Mercado near Durango, Mexico (Manceau *et al.*, 2022 ▸). Using *DFT2FEFFIT*, we show that alternativeCe^3+^ + F^−^ ↔ Ca^2+^ substitution (Ce2–F model) and 2Ce^3+^ + Vac → 3Ca^2+^ substitution (2Ce2–Vac model) are nonfitting models.

## 2. Software details

### 2.1. Input

*DFT2FEFFIT* is open-source code written in Python. It uses a command-line interface, which is invoked with a Python entry point. The user is then prompted to enter the input filename. The following input data are required: the experimental χ function to fit (line 1), the number of scattering paths (*n*, line 2), the *k* weighting of χ for the fit (*k^n^*χ, line 3), the *k* range of the fit (line 4), 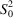 (line 5), whether Δ*E* is adjusted (integer 1) or fixed (integer 0) (line 6), the value of Δ*E* if no variation is allowed (integer 0), or its interval of variation (Δ*E*_min_, Δ*E*_max_) and the step size (line 7), and the list of scattering paths [lines 8 to 8 + (*n* − 1)]. Each path line is structured as follows: a line number (*e.g.* path ID); a string (*e.g.* chemical symbol, SS or MS for single or multiple scattering path); the path distance, only added for easy reference and not actually part of the fit; χ*_i_*; the format of χ*_i_* [*FEFF* format (chip000*n*) or simply two columns, *k*, χ*_i_*]; whether σ*_i_* is optimized (1) or not (0); the initial σ*_i_* value; σ_*i*,min_; σ_*i*,max_; and the path ID with which the σ*_i_* value is co-varied, −1 if the σ values are not linked. Path lines commented with a hash (#) symbol are ignored. At the end of the refinement, the code provides the optimized values, the experimental and calculated *k*-weighted χ functions (ASCII data and plot), the modulus and real part of the Fourier transform (*i.e.* RDF) of *k^n^*χ_exp_ and *k^n^*χ_calc_ using a Kaiser–Bessel window (β = 2.5), and the fit residual expressed as the normalized sum of squared differences [NSS = ∑(*k^n^*χ_exp_ − *k^n^*χ_calc_)^2^/∑(*k^n^*χ_exp_)^2^].

### 2.2. Calculation

The software seeks to minimize NSS by optimizing σ*_i_* for each Δ*E* value. Because χ_calc_ varies nonlinearly with *σ_i_* [equation (1)], the minimization of NSS toward the local minimum is performed iteratively by following the negative of the first derivative of equation (1) with respect to σ_*i*_ (gradient-descent method) at each iteration. The scheme is iterated until NSS reaches a plateau (ΔNSS = 10^−7^) or for a user-defined fixed number of iterations. Convergence is speeded up by rescaling the input σ*_i_* values to the [−1, 1] range according to 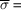 2(σ*_i_* − σ_mean_)/(σ_max_ − σ_min_), with σ_mean_ = 0.5(σ_max_ − σ_min_). Wolfe’s conditions (Wolfe, 1969 ▸; Nocedal & Wright, 2006 ▸) are used to determine the appropriate step size for each line search of strict descent at a point *m_n_* = 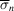. The update to *m_n_* for the next iteration is *m*_*n*+1_ = *m*_*n*_ + α*_n_p_n_*, where α*_n_* is the new step size computed from the line search at *m_n_* to satisfy the Wolfe conditions and *p_n_* is the search direction. The input scripts for the DFT models are deposited in the NOMAD repository (Draxl & Scheffler, 2019 ▸) at https://dx.doi.org/10.17172/NOMAD/2024.02.09-1.

The gradient-descent optimization method was preferred over the Levenberg–Marquardt (LM) method for several reasons. The LM method requires an estimation of the Jacobian of the forward problem in order to build the Gauss–Newton Hessian matrix. This step is not needed with the steepest-descent algorithm. The LM method does not include a line search that would ensure proper convergence (Wolfe’s conditions) and would therefore need to be coupled with Wolfe’s conditions to ensure convergence. Lastly, the LM method requires another tuning parameter for the damping of the diagonal of the Gauss–Newton Hessian matrix.

## 3. Case study

EXAFS spectroscopy probes the local structure of a given element up to about 6 Å. Modeling by DFT the bonding environment of a substituent up to this distance requires optimizing the geometry of rather large clusters comprising more than one hundred atoms. DFT methods exploiting a linear combination of plane waves, as implemented in the *Vienna Ab-initio Simulation Package* (*VASP*; Kresse, 1995 ▸; Kresse & Furthmüller, 1996 ▸), are in this respect more cost effective than methods adopting a linear combination of local atomic orbitals, usually represented as Gaussian-type orbitals, as implemented in *CRYSTAL* (Dovesi *et al.*, 2014 ▸). Comparison of the DFT structures obtained with *VASP* and *CRYSTAL14* on Ce–FAp clusters of 336 atoms (2 × 2 × 2 supercell, radius ≃ 6 Å) showed that *CRYSTAL14* did not provide superior models, even with the accurate PBEsol functional (Perdew *et al.*, 2008 ▸) and basis sets of triple-zeta quality for Ca, P, O and F. Therefore, all optimizations reported in this study were performed with *VASP* to speed up the calculations. Details of the *VASP* parameters and functionals are given in the supporting information.

**Figure 3:**
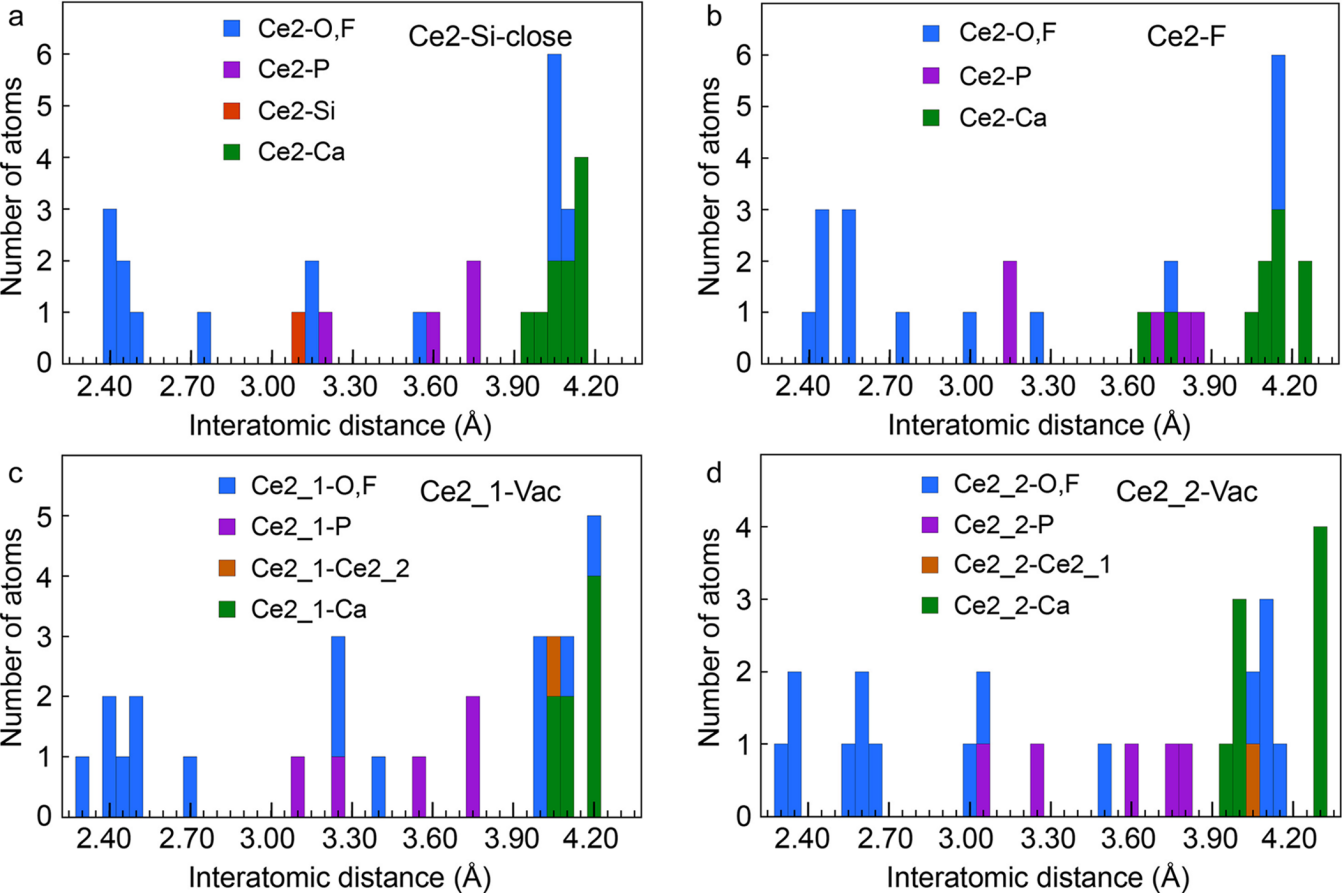
Population histograms of the computed Ce2—(O,F,P,Ca,Ce) distances for (*a*) the Ce2–Si-close model, (*b*) the Ce2–F model and (*c*), (*d*) the two Ce atoms of the 2Ce2–Vac model. Computation details can be found in the supporting information. The number of atoms is counted in intervals of 0.05 Å.

The radial distributions of Ce in the Ce2–Si-close, Ce2–F and 2Ce2–Vac models up to *R* = 4.3 Å are shown in Fig. 3 ▸, and the Cartesian coordinates of the models are listed in the supporting information. The Ce2–F model essentially differs from the optimal Ce2–Si-close model by (i) an increase in coordination from 6 to 7, and hence an increase in the average Ce2—(O,F) distance from 2.43 to 2.48 Å due to the incorporation of the interstitial F atom at 2.42 Å from Ce2, and (ii) the displacement to shorter distance of two Ca atoms [*d*(Ce2—Ca) = 3.65–3.75 Å] and to longer distance of two further Ca atoms [*d*(Ce2—Ca) = 4.26 Å]. Regarding the 2Ce2–Vac model, one Ce atom of the paired Ce atoms (Ce2_1) has a similar local structure to Ce in Ce2–Si-close [Fig. 3 ▸(*c*)], whereas the other Ce atom (Ce2_2) has a distinctive bonding environment characterized by a split of the first (O,F) shell and longer Ce2—Ca distances [Fig. 3 ▸(*d*)].

**Figure 4:**
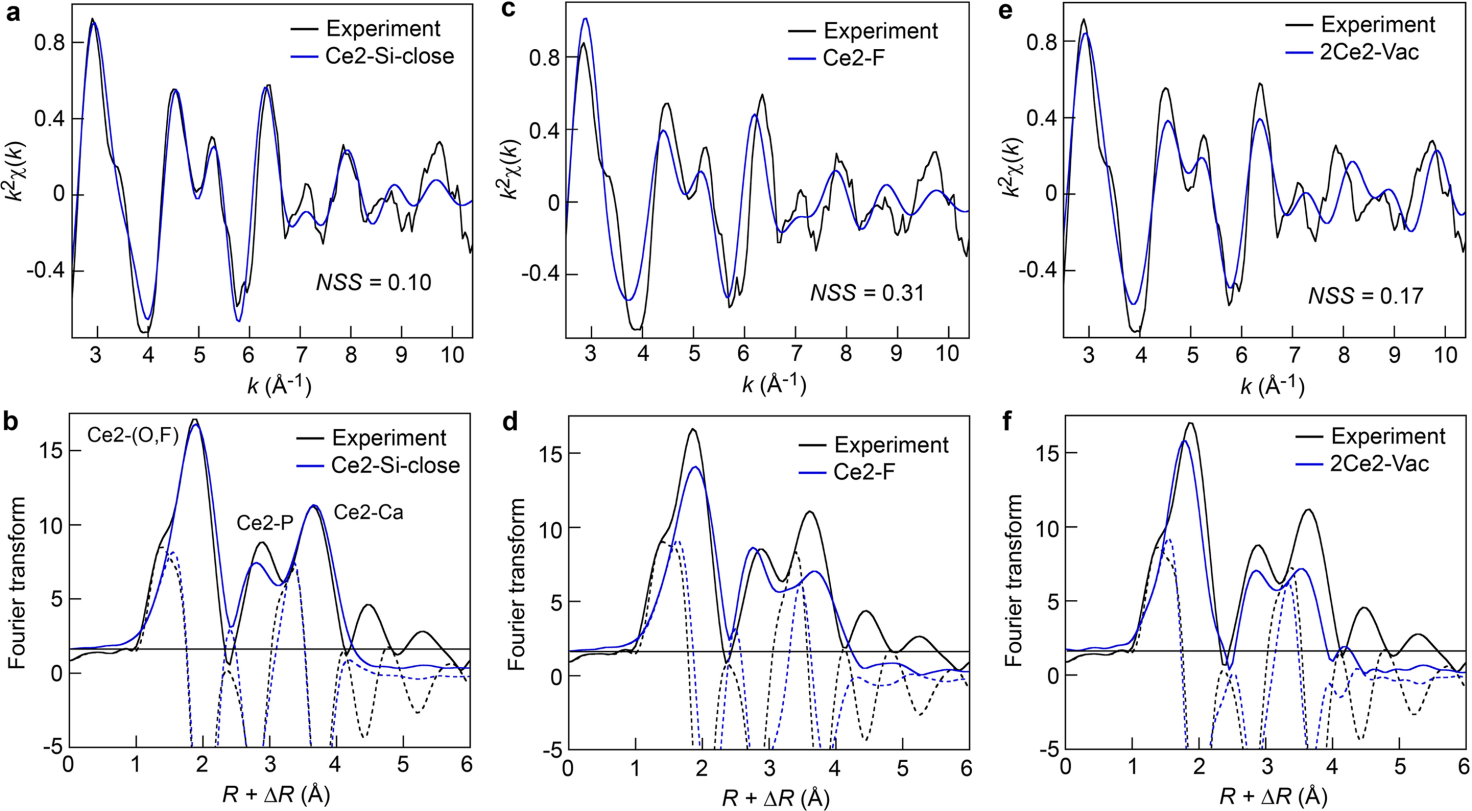
Experimental and DFT-derived theoretical Ce *L*_3_ edge EXAFS spectra and radial distribution functions (magnitude and real part of the *k*^2^-weighted Fourier transform). (*a*), (*b*) Ce2–Si-close model, (*c*), (*d*) Ce2–F model and (*e*), (*f*) 2Ce2–Vac model.

The best-fit results of the Ce *L*_3_ edge EXAFS spectrum for the Durango FAp with the calculated EXAFS spectra for the three DFT models up to *R* = 4.3 Å, together with the corresponding RDF, are shown in Fig. 4 ▸. The data were collected at room temperature on beamline ID24-DCM at the Euro­pean Synchrotron Radiation Facility in high-energy-resolution mode (HERFD-EXAFS) using five analyzer crystals bent to a radius of 0.5 m (Rovezzi *et al.*, 2017 ▸; Glatzel *et al.*, 2021 ▸). Best-fit calculations were conducted by optimizing initially Δ*E* and one σ value for all SS paths (χ_NLEG=2_ in *FEFF*). Afterwards, individual SS paths were grouped into shells (O1, P1, P2, Ca) and their σ*_i_* values refined. The criterion for retaining a new SS path (*i.e.* σ*_i_* value) was that the fit had to improve by at least 5% and be physically meaningful. A single σ value was applied to all MS paths calculated by *FEFF* (χ_NLEG=4_ − χ_NLEG=2_). The optimal Δ*E* value varied marginally (<1 eV) from one fit to another. 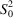 was fixed to 0.9. Best-fit EXAFS parameters of the three DFT models are reported in the supporting information.

Our results show that coupled Ce^3+^ + F^−^ ↔ Ca^2+^ (Ce2–F model) and 2Ce^3+^ + Vac → 3Ca^2+^ (2Ce2–Vac model) are incompatible models. Adding an F atom or removing a Ca atom near a Ce atom are sources of disorder, which manifests on the calculated RDF by a misfit of the Ce2—(O,F) shell and a loss of amplitude of the Ce2—P and Ce2—Ca peaks. Thus, these results underscore the high sensitivity of *DFT2FEFFIT* for detailed characterization of the local structure of elements in complex environments. EXAFS alone does not allow differentiation between P and Si neighbors, because their scattering powers are similar, or the detection of a light F atom and a vacancy site. This distinction becomes possible by comparing the theoretical EXAFS spectra derived from DFT structure models with experiment. *DFT2FEFFIT* may, therefore, be considered as a useful tool for the validation of hypothesis-driven structure models based on EXAFS analysis.

## 4. Availability of *DFT2FEFFIT*

The Python script of *DFT2FEFFIT* is available online at https://gitlab.esrf.fr/scientific-software/dft2feffit and in thesupporting information.

## 5. Related literature

For further literature related to the supporting information, see Blöchl (1994 ▸), Gautier *et al.* (2015 ▸), Gonthier *et al.* (2012 ▸), Kresse & Joubert (1999 ▸), Perdew *et al.* (1996 ▸) and Steinmann & Corminboeuf (2011 ▸).

## Supplementary Material

Details of DFT calculations, input scripts, EXAFS spectrum, Cartesian coordinates of the DFT models. DOI: 10.1107/S1600576724005454/vb5074sup1.pdf

Python code. DOI: 10.1107/S1600576724005454/vb5074sup2.zip

## References

[bb1] Ankudinov, A. L. & Rehr, J. J. (1997). *Phys. Rev. B*, **56**, R1712–R1716.

[bb101] Blöchl, P. E. (1994). *Phys. Rev. B*, **50**, 17953–17979. 10.1103/physrevb.50.17953 9976227

[bb2] Boyanov, B. I., Bunker, G. & Morrison, T. I. (1996). *J. Synchrotron Rad.* **3**, 120–128. 10.1107/S0909049596003585 16702669

[bb3] Chantler, C. T., Boscherini, F. & Bunker, B. (2020). Editors. *International Tables for Crystallography*, Vol. I, *X-ray Absorption Spectroscopy and Related Techniques*, 1st online ed. Chester: IUCr.

[bb4] Cotelesage, J. J. H., Pushie, M. J., Grochulski, P., Pickering, I. J. & George, G. N. (2012). *J. Inorg. Biochem.* **115**, 127–137. 10.1016/j.jinorgbio.2012.06.019 22824156

[bb5] Crozier, E. D. (1997). *Nucl. Instrum. Methods Phys. Res. B*, **133**, 134–144.

[bb6] Dalba, G. & Fornasini, P. (1997). *J. Synchrotron Rad.* **4**, 243–255. 10.1107/S0909049597006900 16699237

[bb8] Dovesi, R., Orlando, R., Erba, A., Zicovich–Wilson, C. M., Civalleri, B., Casassa, S., Maschio, L., Ferrabone, M., De La Pierre, M., D’Arco, P., Noël, Y., Causà, M., Rérat, M. & Kirtman, B. (2014). *Int. J. Quantum Chem.* **114**, 1287–1317.

[bb9] Draxl, C. & Scheffler, M. (2019). *J. Phys. Mater.* **2**, 036001.

[bb10] Fleet, M., Liu, X. & Pan, Y. (2000). *J. Solid State Chem.* **149**, 391–398.

[bb102] Gautier, S., Steinmann, S., Michel, C., Fleurat-Lessard, P. & Sautet, P. (2015). *Phys. Chem. Chem. Phys.* **17**, 28921–28930. 10.1039/c5cp04534g 26455444

[bb11] Glatzel, P., Harris, A., Marion, P., Sikora, M., Weng, T.-C., Guilloud, C., Lafuerza, S., Rovezzi, M., Detlefs, B. & Ducotté, L. (2021). *J. Synchrotron Rad.* **28**, 362–371. 10.1107/S1600577520015416 33399588

[bb108] Gonthier, J. F., Steinmann, S. N., Wodrich, M. D. & Corminboeuf, C. (2012). *Chem. Soc. Rev.* **41**, 4671–4687. 10.1039/c2cs35037h 22660008

[bb12] Harlov, D. E. & Rakovan, J. F. (2015). Guest editors. *Apatite: A Mineral for All Seasons.**Elements*, Vol. 11, No. 3. Washington, DC: Mineralogical Society of America.

[bb13] Harris, H. H., George, G. N. & Rajagopalan, K. V. (2006). *Inorg. Chem.* **45**, 493–495. 10.1021/ic0512274 16411679

[bb14] Hughes, J. M., Cameron, M. & Crowley, K. D. (1989). *Am. Miner.* **74**, 870–876.

[bb103] Kresse, G. (1995). *J. Non-Cryst. Solids*, **193**, 2222–2229.

[bb104] Kresse, G. & Furthmüller, J. (1996). *Comput. Mater. Sci.* **6**, 15–50.

[bb105] Kresse, G. & Joubert, D. (1999). *Phys. Rev. B*, **59**, 1758–1775.

[bb15] Manceau, A., Mathon, O., Lomachenko, K. A., Rovezzi, M., Kvashnina, K. O., Boiron, M. C., Brossier, R. & Steinmann, S. N. (2024). *ACS Earth Space Chem.* **8**, 119–128.

[bb16] Manceau, A., Paul, S., Simionovici, A., Magnin, V., Balvay, M., Findling, N., Rovezzi, M., Muller, S., Garbe-Schönberg, D. & Koschinsky, A. (2022). *ACS Earth Space Chem.* **6**, 2093–2103.

[bb17] Marcus, M. A., Chen, H. S., Espinosa, G. P. & Tsai, C. L. (1986). *Solid State Commun.* **58**, 227–230.

[bb18] Nocedal, J. & Wright, S. J. (2006). *Numerical Optimization*. New York: Springer.

[bb106] Perdew, J. P., Burke, K. & Ernzerhof, M. (1996). *Phys. Rev. Lett.* **77**, 3865–3868. 10.1103/PhysRevLett.77.3865 10062328

[bb19] Perdew, J. P., Ruzsinszky, A., Csonka, G. I., Vydrov, O. A., Scuseria, G. E., Constantin, L. A., Zhou, X. L. & Burke, K. (2008). *Phys. Rev. Lett.* **100**, 136406. 10.1103/PhysRevLett.100.136406 18517979

[bb20] Rehr, J. J. & Albers, R. C. (1990). *Phys. Rev. B*, **41**, 8139–8149. 10.1103/physrevb.41.8139 9993134

[bb21] Rehr, J. J. & Albers, R. C. (2000). *Rev. Mod. Phys.* **72**, 621–654.

[bb22] Rehr, J. J., Kas, J. J. & Vila, F. D. (2020). *EXAFS: Theory and Approaches*. In *International Tables for Crystallography*, Vol. I, *X-ray Absorption Spectroscopy and Related Techniques*, 1st online ed. Chester: IUCr.

[bb23] Rønsbo, J. G. (1989). *Am. Miner.* **74**, 896–901.

[bb24] Rovezzi, M., Lapras, C., Manceau, A., Glatzel, P. & Verbeni, R. (2017). *Rev. Sci. Instrum.* **88**, 013108. 10.1063/1.4974100 28147645

[bb107] Steinmann, S. N. & Corminboeuf, C. (2011). *J. Chem. Theory Comput.* **7**, 3567–3577. 10.1021/ct200602x 26598255

[bb25] Stern, E. A., Sayers, D. E. & Lytle, F. W. (1975). *Phys. Rev. B*, **11**, 4836–4846.

[bb26] Terry, J., Lau, M. L., Sun, J. T., Xu, C., Hendricks, B., Kise, J., Lnu, M., Bagade, S., Shah, S., Makhijani, P., Karantha, A., Boltz, T., Oellien, M., Adas, M., Argamon, S., Long, M. & Guillen, D. P. (2021). *Appl. Surf. Sci.* **547**, 149059.

[bb27] Timoshenko, J., Wrasman, C., Luneau, M., Shirman, T., Cargnello, M., Bare, S., Aizenberg, J., Friend, C. & Frenkel, A. (2019). *Nano Lett.* **19**, 520–529. 10.1021/acs.nanolett.8b04461 30501196

[bb28] Wolfe, P. (1969). *SIAM Rev.* **11**, 226–235.

