# Supplementary material for: DFT2FEFFIT: a density-functional-theory-based structural toolkit to analyze EXAFS spectra
Source: J Appl Crystallogr. 2024 Jul 17;57(Pt 4):1229–34. doi: 10.1107/S1600576724005454 (PMC11299619; doi:10.1107/S1600576724005454)
Supplement: Supplementary file 1 [file j-57-01229-sup1.pdf]

JOURNAL OF
APPLIED
CRYSTALLOGRAPHY

Volume 57 (2024)

Supporting information for article:

DFT2FEFFIT: a DFT-based structural toolkit to analyze EXAFS spectra

Alain Manceau, Romain Brossier, Olivier Mathon, Kirill A. Lomachenko, Marius Retegan, Pieter Glatzel and Stephan N. Steinmann

S1. DFT calculation

DFT computations were performed in the Vienna Ab-initio Simulation Package (VASP) software (Kresse, 1995; Kresse & Furthmuller, 1996). The projector augmented wave (PAW) pseudopotentials (Blochl, 1994; Kresse & Joubert, 1999) were used for the core-electron interaction, in combination with a converged energy cutoff of 500 eV for the planewave basis set. The energies and forces were evaluated at the generalized gradient approximation (GGA) with a Perdew-Burke-Ernzerhof (PBE; Perdew *et al.*, 1996) exchange-correlation functional in conjunction with the density-dependent dispersion correction dDsC (Steinmann & Corminboeuf, 2011; Gautier *et al.*, 2015). The Brillouin zone of 2 x 2 x 2 supercells with stoichiometry $Ca_{80}P_{48}O_{192}F_{16}$ (FAp has two formula units in the primitive cell) and cell vector lengths of about 18.8 Å and 13.7 Å for in-plane and out-of-plane directions was probed at the Gamma point. A Fermi-smearing of 0.026 eV (~300 K) was applied, but had no impact on the electronic energy for this insulating material with a band gap of at least 4 eV. During the self-consistent field procedure, the wave functions were optimized to an energy-change below 10^{-6} eV. The numerical settings for the fast Fourier transformation grids was set to “accurate” and the real-space projector operators were determined via the recommended automatic optimization scheme. For geometry optimizations all atoms and the unit cell were fully relaxed until the maximum force was below 0.05 eV/Å. The atomic Hirshfeld charges, which are known to depend little on the electronic structure and the basis set and are well suited for assessing charge-transfers (Gonthier *et al.*, 2012), have been taken from the standard output of the dDsC dispersion correction. The maximum error in the interatomic distances of FAp calculated with VASP relative to the diffraction values (Hughes *et al.*, 1989) is 1.15% (Manceau *et al.*, 2024). The starting configurations of the substitutional models are produced by replacing one element by another in the FAp structure. No symmetry constraints were applied, VASP guesses an initial symmetry based on the initial coordinates and unit cell.

S2. Input script and best-fit parameters of the Ce2-Si-close model

Input :

```
Ce-EXAFS_Durango_RT.chi
22
2
2.5 10.4
0.9
```

1
-2.5 -0.5 0.3
1; F; 2.379; chip0001.dat;FEFF;1;0.06; 0.04; 0.13; 2
2; O; 2.415; chip0002.dat; FEFF; 1; 0.06; 0.04; 0.13; -1
3; O; 2.416; chip0003.dat; FEFF; 1; 0.06; 0.04; 0.13; 2
4; O; 2.436; chip0004.dat; FEFF; 1; 0.06; 0.04; 0.13; 2
5; O; 2.437; chip0005.dat; FEFF; 1; 0.06; 0.04; 0.13; 2
6; O; 2.487; chip0006.dat; FEFF; 1; 0.06; 0.04; 0.13; 2
7; O; 2.754; chip0007.dat; FEFF; 1; 0.06; 0.04; 0.13; 2
8; Si; 3.095; chip0008.dat; FEFF; 1; 0.06; 0.04; 0.13; -1
11; P; 3.197; chip0011.dat; FEFF; 1; 0.06; 0.04; 0.13; 8
13; P; 3.616; chip0013.dat; FEFF; 1; 0.06; 0.04; 0.13; -1
14; 2P; 3.774; chip0014.dat; FEFF; 1; 0.06; 0.04; 0.13; 13
15; Ca; 3.967; chip0015.dat; FEFF; 1; 0.06; 0.04; 0.13; -1
16; Ca; 4.010; chip0016.dat; FEFF; 1; 0.06; 0.04; 0.13; 15
20; Ca; 4.066; chip0020.dat; FEFF; 1; 0.06; 0.04; 0.13; 15
21; Ca; 4.068; chip0021.dat; FEFF; 1; 0.06; 0.04; 0.13; 15
24; Ca; 4.100; chip0024.dat; FEFF; 1; 0.06; 0.04; 0.13; 15
25; Ca; 4.101; chip0025.dat; FEFF; 1; 0.06; 0.04; 0.13; 15
26; Ca; 4.131; chip0026.dat; FEFF; 1; 0.06; 0.04; 0.13; 15
27; Ca; 4.133; chip0027.dat; FEFF; 1; 0.06; 0.04; 0.13; 15
28; Ca; 4.144; chip0028.dat; FEFF; 1; 0.06; 0.04; 0.13; 15
29; Ca; 4.148; chip0029.dat; FEFF; 1; 0.06; 0.04; 0.13; 15
30; MS; 4.000; Ce2-Si-close_MS.chi; 2C; 1; 0.10; 0.08; 0.17; -1

Best-fit parameters:
ΔE = -1.0 eV; σ(O,F) = 0.12 Å; σ(Si,P1) = 0.07 Å; σ(P2) = 0.12 Å; σ(Ca) = 0.11 Å; ; σ(MS) = 0.17 Å; *NSS* = 0.10
The σ values of the cation shells at 8-12 K are: σ(Si,P1) = 0.04 Å; σ(P2) = 0.09 Å; σ(Ca) = 0.08 Å (Manceau *et al.*, 2024).

S3. Input script and best-fit parameters of the Ce2-F model

Input :
Ce-EXAFS_Durango_RT.chi
24
2
2.5 10.4
0.9
1
-2.5 -0.5 0.3
1; F;2.31;chip0001.dat;FEFF;1;0.06; 0.04; 0.13; 2
2; O;2.31;chip0002.dat;FEFF;1; 0.06;0.04; 0.13; -1
3; O;2.31;chip0003.dat;FEFF;1; 0.06;0.04; 0.13; 2
4; O;2.31;chip0004.dat;FEFF;1; 0.06;0.04; 0.13; 2
5; O;2.31;chip0005.dat;FEFF;1; 0.06;0.04; 0.13; 2
6; O;2.31;chip0006.dat;FEFF;1; 0.06;0.04; 0.13; 2
7; O;2.31;chip0007.dat;FEFF;1; 0.06;0.04; 0.13; 2
8; O;2.31;chip0008.dat;FEFF;1; 0.06;0.04; 0.13; 2
10; P;2.31;chip0010.dat;FEFF;1; 0.06;0.04; 0.13; -1

11; P;2.31;chip0011.dat;FEFF;1; 0.06; 0.04; 0.13;10
14; P;2.31;chip0014.dat;FEFF;1; 0.06; 0.04; 0.13; -1
17; P;2.31;chip0017.dat;FEFF;1;0.06; 0.04; 0.13; 14
18; P;2.31;chip0018.dat;FEFF;1;0.06; 0.04; 0.13; 14
13; Ca;2.31;chip0013.dat;FEFF;1;0.06; 0.04; 0.15; -1
16; Ca;2.31;chip0016.dat;FEFF;1;0.06; 0.04; 0.15; 13
19; Ca;2.31;chip0019.dat;FEFF;1;0.06; 0.04; 0.15; 13
20; Ca;2.31;chip0020.dat;FEFF;1;0.06; 0.04; 0.15; 13
21; Ca;2.31;chip0021.dat;FEFF;1;0.06; 0.04; 0.15; 13
23; Ca;2.31;chip0023.dat;FEFF;1;0.06; 0.04; 0.15; 13
24; Ca;2.31;chip0024.dat;FEFF;1;0.06; 0.04; 0.15; 13
27; Ca;2.31;chip0027.dat;FEFF;1;0.06; 0.04; 0.15; 13
28; Ca;2.31;chip0028.dat;FEFF;1;0.06; 0.04; 0.15; 13
29; Ca;2.31;chip0029.dat;FEFF;1;0.06; 0.04; 0.15; 13
30; F;2.31;Ce2_F_interstitiel_MS.dat;2C;1;0.10; 0.08; 0.17; -1

Best-fit parameters:
ΔE = -2.5 eV; σ(O,F) = 0.13 Å; σ(P1) = 0.08 Å; σ(P2) = 0.13 Å; σ(Ca) = 0.12 Å; ; σ(MS) = 0.17 Å; NSS = 0.31

S4. Input script and best-fit parameters of the 2Ce2-Vac model

The amplitude of the Durango spectrum was multiplied by two to fit the DFT spectra at the Ce2_1-Vac and Ce2_2-Vac sites, and afterward the experimental and optimized spectra were divided by two.

Input:
Ce-EXAFS_Durango_RTx2.chi
44
2
2.5 10.4
0.9
1
-2.5 -0.5 0.3
#Ce2-1-Vac site
1; F;2.31;chip0001_1.dat;FEFF;1;0.06; 0.04; 0.13; 2
2; O;2.31;chip0002_1.dat;FEFF;1; 0.06;0.04; 0.13; -1
3; O;2.31;chip0003_1.dat;FEFF;1; 0.06;0.04; 0.13; 2
4; O;2.31;chip0004_1.dat;FEFF;1; 0.06;0.04; 0.13; 2
5; O;2.31;chip0005_1.dat;FEFF;1; 0.06;0.04; 0.13; 2
6; O;2.31;chip0006_1.dat;FEFF;1; 0.06;0.04; 0.13; 2
7; O;2.31;chip0007_1.dat;FEFF;1; 0.06;0.04; 0.13; 2
8; P;2.31;chip0008_1.dat;FEFF;1; 0.06;0.04; 0.13; -1
9; P;2.31;chip0009_1.dat;FEFF;1; 0.06; 0.04; 0.13;8
13; P;2.31;chip0013_1.dat;FEFF;1; 0.06; 0.04; 0.13; -1
14; P;2.31;chip0014_1.dat;FEFF;1;0.06; 0.04; 0.13; 13
15; P;2.31;chip0015_1.dat;FEFF;1;0.06; 0.04; 0.13; 13
19; Ca;2.31;chip0019_1.dat;FEFF;1;0.06; 0.04; 0.15; -1
20; Ca;2.31;chip0020_1.dat;FEFF;1;0.06; 0.04; 0.15; 19
21; Ce2;2.31;chip0021_1.dat;FEFF;1;0.06; 0.04; 0.15; -1
23; Ca;2.31;chip0023_1.dat;FEFF;1;0.06; 0.04; 0.15; 19

24; Ca;2.31;chip0024_1.dat;FEFF;1;0.06; 0.04; 0.15; 19
25; Ca;2.31;chip0025_1.dat;FEFF;1;0.06; 0.04; 0.15; 19
26; Ca;2.31;chip0026_1.dat;FEFF;1;0.06; 0.04; 0.15; 19
28; Ca;2.31;chip0028_1.dat;FEFF;1;0.06; 0.04; 0.15; 19
29; Ca;2.31;chip0029_1.dat;FEFF;1;0.06; 0.04; 0.15; 19
30; MS;2.31;Ce2_1_Vac_Together_MS.chi;2C;1;0.10; 0.08; 0.17; -1
#Ce2-2-Vac site
31; F;2.31;chip0001_2.dat;FEFF;1;0.06; 0.04; 0.13; 2
32; O;2.31;chip0002_2.dat;FEFF;1; 0.06;0.04; 0.13; 2
33; O;2.31;chip0003_2.dat;FEFF;1; 0.06;0.04; 0.13; 2
34; O;2.31;chip0004_2.dat;FEFF;1; 0.06;0.04; 0.13; 2
35; O;2.31;chip0005_2.dat;FEFF;1; 0.06;0.04; 0.13; 2
36; O;2.31;chip0006_2.dat;FEFF;1; 0.06;0.04; 0.13; 2
37; O;2.31;chip0007_2.dat;FEFF;1; 0.06;0.04; 0.13; 2
38; P;2.31;chip0010_2.dat;FEFF;1; 0.06;0.04; 0.13; 8
39; P;2.31;chip0011_2.dat;FEFF;1; 0.06; 0.04; 0.13;8
40; P;2.31;chip0013_2.dat;FEFF;1; 0.06; 0.04; 0.13; 13
41; P;2.31;chip0014_2.dat;FEFF;1;0.06; 0.04; 0.13; 13
42; P;2.31;chip0015_2.dat;FEFF;1;0.06; 0.04; 0.13; 13
43; Ca;2.31;chip0016_2.dat;FEFF;1;0.06; 0.04; 0.15; 19
44; Ca;2.31;chip0017_2.dat;FEFF;1;0.06; 0.04; 0.15; 19
45; Ca;2.31;chip0018_2.dat;FEFF;1;0.06; 0.04; 0.15; 19
46; Ca;2.31;chip0019_2.dat;FEFF;1;0.06; 0.04; 0.15; 19
47; Ce1;2.31;chip0021_2.dat;FEFF;1;0.06; 0.04; 0.15; 21
48; Ca;2.31;chip0026_2.dat;FEFF;1;0.06; 0.04; 0.15; 19
49; Ca;2.31;chip0027_2.dat;FEFF;1;0.06; 0.04; 0.15; 19
50; Ca;2.31;chip0028_2.dat;FEFF;1;0.06; 0.04; 0.15; 19
51; Ca;2.31;chip0029_2.dat;FEFF;1;0.06; 0.04; 0.15; 19
52; MS;2.31;Ce2_2_Vac_Together_MS.chi;2C;1;0.10; 0.08; 0.17; 30

Best-fit parameters:

ΔE = -1.3 eV; σ(O,F) = 0.05 Å; σ(P1) = 0.04 Å; σ(P2) = 0.08 Å; σ(Ca) = 0.07 Å; ; σ(MS) = 0.17 Å; *NSS* = 0.17

S5. References

Blochl, P. E. (1994). *Phys. Rev. B.* **50**, 17953–17979.

Gautier, S., Steinmann, S., Michel, C., Fleurat-Lessard, P. & Sautet, P. (2015). *Phys. Chem. Chem. Phys.* **17**, 28921–28930.

Gonthier, J. F., Steinmann, S. N., Wodrich, M. D. & Corminboeuf, C. (2012). *Chem. Soc. Rev.* **41**, 4671–4687.

Hughes, J. M., Cameron, M. & Crowley, K. D. (1989). *Am. Miner.* **74**, 870–876.

Kresse, G. (1995). *J. Non-Cryst. Solids* **193**, 222–229.

Kresse, G. & Furthmuller, J. (1996). *Comput. Mater. Sci.* **6**, 15–50.

Kresse, G. & Joubert, D. (1999). *Phys. Rev. B.* **59**, 1758–1775.

Manceau, A., Mathon, O., Lomachenko, K. A., Rovezzi, M., Kvashnina, K. O., Boiron, M. C., Brossier, R. & Steinmann, S. N. (2024). *ACS Earth Space Chem.* **8**, 119–128.

Perdew, J. P., Burke, K. & Ernzerhof, M. (1996). *Phys. Rev. Lett.* **77**, 3865–3868.

Steinmann, S. N. & Corminboeuf, C. (2011). *J. Chem. Theory Comput.* **7**, 3567–3577.

S6. EXAFS spectrum (Ce-EXAFS_Durango_RT.chi)

2.0000	0.033285
2.0500	-0.0056294
2.1000	-0.063230
2.1500	-0.10729
2.2000	-0.13473
2.2500	-0.15114
2.3000	-0.15464
2.3500	-0.15564
2.4000	-0.14836
2.4500	-0.13583
2.5000	-0.11378
2.5500	-0.087390
2.6000	-0.052464
2.6500	-0.011952
2.7000	0.024822
2.7500	0.057343
2.8000	0.080193
2.8500	0.098267
2.9000	0.10792
2.9500	0.11015
3.0000	0.10162
3.0500	0.087279
3.1000	0.073539
3.1500	0.052451
3.2000	0.037923
3.2500	0.024309
3.3000	0.019305
3.3500	0.015608
3.4000	0.013077
3.4500	0.012445
3.5000	0.0076357
3.5500	0.0012066
3.6000	-0.0064417
3.6500	-0.017532
3.7000	-0.028536
3.7500	-0.037126
3.8000	-0.043871
3.8500	-0.048288
3.9000	-0.048011
3.9500	-0.047283
4.0000	-0.045498
4.0500	-0.044836
4.1000	-0.039719
4.1500	-0.034825
4.2000	-0.024371
4.2500	-0.011284
4.3000	0.0011470
4.3500	0.010359
4.4000	0.020198

4.4500	0.026280
4.5000	0.027212
4.5500	0.027500
4.6000	0.026110
4.6500	0.022123
4.7000	0.017449
4.7500	0.013199
4.8000	0.010161
4.8500	0.0062488
4.9000	0.0027761
4.9500	0.00062829
5.0000	0.00065193
5.0500	0.0018544
5.1000	0.0012388
5.1500	0.0054323
5.2000	0.0087760
5.2500	0.010786
5.3000	0.011342
5.3500	0.0094710
5.4000	0.0063844
5.4500	-0.00050645
5.5000	-0.0033045
5.5500	-0.0057661
5.6000	-0.0051372
5.6500	-0.0091065
5.7000	-0.012307
5.7500	-0.017990
5.8000	-0.017328
5.8500	-0.016017
5.9000	-0.011794
5.9500	-0.014823
6.0000	-0.014039
6.0500	-0.011419
6.1000	-0.0075582
6.1500	-0.00086814
6.2000	0.0060930
6.2500	0.0098138
6.3000	0.012384
6.3500	0.013952
6.4000	0.014752
6.4500	0.013128
6.5000	0.010026
6.5500	0.0084722
6.6000	0.0056942
6.6500	0.00031908
6.7000	-0.0051293
6.7500	-0.0049745
6.8000	-0.0030537
6.8500	-0.0021290
6.9000	-0.0022931

6.9500	-0.0019634
7.0000	-0.0010604
7.0500	-0.00088457
7.1000	0.0010552
7.1500	0.0013429
7.2000	0.00062010
7.2500	0.00053089
7.3000	-0.0028961
7.3500	-0.0037367
7.4000	-0.0028782
7.4500	-0.0051570
7.5000	-0.0035957
7.5500	-0.0024387
7.6000	-0.00025628
7.6500	-0.00041177
7.7000	-0.00082858
7.7500	0.0021290
7.8000	0.0026348
7.8500	0.0047353
7.9000	0.0033215
7.9500	0.0036446
8.0000	0.0029286
8.0500	0.0025423
8.1000	0.00099688
8.1500	0.0020861
8.2000	-0.00078354
8.2500	-0.0018291
8.3000	-0.0023871
8.3500	-0.0012933
8.4000	-0.0015607
8.4500	-0.00083020
8.5000	-0.00079647
8.5500	-0.00045056
8.6000	-0.00026179
8.6500	-0.00021707
8.7000	-0.00041447
8.7500	-0.00056862
8.8000	0.00065174
8.8500	-0.00058880
8.9000	-0.00033076
8.9500	-0.0012026
9.0000	-0.0023989
9.0500	-0.0022052
9.1000	-0.0016627
9.1500	-0.00050149
9.2000	-0.0026013
9.2500	-0.0014177
9.3000	-0.00092885
9.3500	-0.00040318
9.4000	0.00059581

9.4500	0.0017088
9.5000	0.0011485
9.5500	0.0016593
9.6000	0.0023186
9.6500	0.0021849
9.7000	0.0027773
9.7500	0.0029719
9.8000	0.0028724
9.8500	0.0021606
9.9000	0.0014000
9.9500	-0.00012312
10.000	0.00036067
10.050	-0.00043315
10.100	0.00054729
10.150	0.00031970
10.200	-0.0023175
10.250	-0.0018420
10.300	-0.0018910
10.350	-0.0028566
10.400	-0.0027410
10.450	-0.0013883

S7. Cartesian coordinates of the DFT models (*a*= 100 Å, *b* = 100 Å, *c* = 100 Å)

S7.1. Ce2-Si-close

58	5.734951401	10.098389649	5.142576935
14	8.283720557	8.342271684	5.144817405
20	9.370090676	5.424408519	3.448190122
20	9.376116308	5.424321125	6.826188073
20	5.958904535	6.095079503	5.145518040
20	4.687826819	2.698758031	13.750127248
20	4.693408561	2.713379275	6.876911998
20	14.086877586	2.719733768	13.755360374
20	14.078150039	2.725585789	6.871931734
20	-0.004483200	10.854971840	13.754304664
20	-0.055058312	10.868976580	6.878993205
20	9.401377178	10.860903145	13.756694287
20	9.397537895	10.785581731	6.854300521
20	0.014423557	5.426747556	6.855233109
20	-0.002612375	5.427625894	13.731330630
20	9.392324608	5.425238117	13.738868836
20	-4.703039008	13.585914353	6.853416076
20	-4.701550119	13.570243142	13.731485624
20	4.693974238	13.627197860	6.872893959
20	4.679385812	13.595096052	13.718583764
20	8.208381147	1.969455574	1.698607485
20	8.207820740	1.970717912	8.587511429
20	17.598283938	1.985559755	1.700613090
20	17.598676748	1.986564493	8.585190103
20	3.502918287	10.137168770	1.666610328
20	3.503040495	10.139490917	8.620606575

20	12.895954836	10.115763145	1.705875617
20	12.895404060	10.115265608	8.579450927
20	3.592378081	6.131338424	1.715461938
20	3.593729285	6.132664897	8.571564789
20	12.964795464	6.122156302	1.705056385
20	12.965728158	6.122524923	8.581146015
20	-1.125533982	14.271412801	1.699183832
20	-1.126363498	14.271457584	8.586478453
20	8.282877764	14.286484203	1.695790928
20	8.281967283	14.286790469	8.587512083
20	1.111147533	2.012214701	5.142229079
20	1.117317294	2.019883362	12.023269433
20	10.498734633	1.998958040	5.143006728
20	10.502392582	2.020479641	12.023694814
20	-3.608801400	10.146471479	5.143128709
20	-3.584417612	10.155539142	12.023784740
20	5.829724306	10.152704110	12.024821781
20	2.299798305	0.033865710	1.703537355
20	2.299095751	0.033813641	8.582291263
20	11.679769298	0.050821382	1.700912110
20	11.678379959	0.050974113	8.583781769
20	-2.400209037	8.180037214	1.699521768
20	-2.399993643	8.180397856	8.584887906
20	7.005698233	8.159906932	1.706976495
20	7.007247986	8.159851094	8.582361993
20	2.333618655	8.058570229	5.143412125
20	2.394066657	8.105678038	12.024728846
20	11.744980291	8.124179967	5.142734421
20	11.778745130	8.102223051	12.023348118
20	-2.311741993	16.228621618	5.143044527
20	-2.303845052	16.251380042	12.023506532
20	7.089237382	16.262794899	5.142181351
20	7.092663027	16.260054547	12.023778017
20	-3.522024580	6.161214078	5.142379071
20	-3.516550635	6.165807477	12.023822169
20	5.877344733	6.160489197	12.024338781
20	-8.206858611	14.312818189	5.142718458
20	-8.207718599	14.312494268	12.023460623
20	1.188438672	14.286001500	5.141985182
20	1.170354424	14.317818613	12.023708919
20	0.015753271	5.429512925	3.419700330
20	-0.001450695	5.430764184	10.304500599
20	9.389872324	5.423139910	10.298130766
20	-4.703908823	13.583478842	3.421676630
20	-4.703012047	13.568593929	10.304434465
20	4.694156830	13.625243939	3.402250020
20	4.680280542	13.593640137	10.317007058
20	4.693874425	2.709986729	3.417361005
20	4.688944464	2.698799826	10.306511351
20	14.077687079	2.726605634	3.422587932

20	14.086177419	2.718887402	10.301044186
20	-0.057583087	10.868551365	3.415022847
20	-0.004861943	10.854874059	10.302402438
20	9.400025001	10.788047212	3.439834151
20	9.401111835	10.864171899	10.299807744
15	1.593556942	3.249957713	1.698609150
15	1.594174964	3.250597214	8.585199357
15	10.981621107	3.236108127	1.700987992
15	-3.113852272	11.386291371	1.696416922
15	-3.113639775	11.385621730	8.587491742
15	6.298935807	11.432346872	1.657619637
15	6.297922609	11.433065262	8.627658160
15	3.121402886	4.871303659	5.144758518
15	3.102449252	4.913612448	12.024474613
15	12.494870077	4.888480456	5.143014920
15	12.494766112	4.888194055	12.024613858
15	-1.598775325	13.035054179	5.143629922
15	-1.598132764	13.040015074	12.024041315
15	7.794049718	13.071192034	5.142867346
15	7.799087546	13.052104237	12.023549962
15	1.097716357	7.907654937	1.708305023
15	1.097976446	7.907301613	8.576158763
15	10.489814252	7.888091920	1.697987276
15	10.489852920	7.888175351	8.586822062
15	-3.606799987	16.039373642	1.697425826
15	-3.608117324	16.038983844	8.586699983
15	5.785904736	16.062409308	1.698273974
15	5.785554161	16.061709157	8.585577848
15	3.596372196	0.243111739	5.143507247
15	3.593371941	0.237504864	12.024227243
15	12.993994593	0.252159736	5.142777067
15	12.982761335	0.249139775	12.023813036
15	10.982361059	3.236756575	8.583035280
15	-1.084091654	8.398747146	5.143180789
15	-1.096499439	8.386343563	12.024495239
15	8.304431640	8.389038642	12.025512934
15	-2.680701452	5.148364809	1.704246801
15	-2.681672325	5.148703294	8.579779543
15	6.699795406	5.120845294	1.681138299
15	6.701340678	5.119968389	8.605417136
15	-7.383494177	13.281861066	1.708182500
15	-7.384456943	13.281648099	8.575193676
15	2.004366390	13.289832581	1.700434952
15	2.003099512	13.290755531	8.582651084
15	16.781018560	3.004732916	5.143223344
15	7.373404367	2.999353564	12.025408983
15	7.400498332	2.948548694	5.144320121
15	16.770843676	3.005707426	12.023763584
15	2.710081028	11.133494263	5.144326361
15	2.669644252	11.159977588	12.025027459

15	12.077112630	11.161035999	5.143708602
15	12.075820863	11.143438305	12.024246554
8	3.018069601	2.649967267	1.702671589
8	3.018799752	2.651098242	8.587929134
8	12.415550393	2.653803323	1.698639416
8	12.415632152	2.653081819	8.592780681
8	-1.681113283	10.800563039	1.692188622
8	-1.680352711	10.801466595	8.598867495
8	7.720049632	10.844726662	1.685793833
8	7.719168176	10.845590114	8.608402644
8	1.697770231	5.481724153	5.144029486
8	1.669847103	5.500004884	12.022530235
8	11.072204720	5.486681826	5.139117718
8	11.062660431	5.467098986	12.021783457
8	-3.013121705	13.653801100	5.141600271
8	-3.028828373	13.625168429	12.021416831
8	6.342789223	13.606894107	5.140678159
8	6.367245203	13.639995365	12.020680807
8	5.580000497	1.281853652	1.705333690
8	5.579023330	1.281397038	8.585400160
8	14.979719618	1.305798051	1.714104284
8	14.980173037	1.305271259	8.577840155
8	0.897597349	9.442956434	1.736139838
8	0.896980883	9.442550664	8.557585110
8	10.276436152	9.420504557	1.691600609
8	10.275760227	9.420505109	8.600367800
8	-0.847120782	6.871837917	5.141239745
8	-0.886742137	6.854914075	12.022082194
8	8.446305880	6.725165927	5.143786763
8	8.525193048	6.860064305	12.023809214
8	-5.581479706	15.000863020	5.141408534
8	-5.598523193	14.993466128	12.022023090
8	3.812312311	15.008611663	5.140025804
8	3.793497206	15.004017268	12.020693654
8	5.480943929	4.169120517	1.677248747
8	5.483689702	4.167171655	8.618905011
8	14.879954075	4.196381027	1.707576545
8	14.879151057	4.196414711	8.583484648
8	0.788991372	12.333454471	1.710789561
8	0.788503979	12.333342971	8.579139377
8	10.180431509	12.327052367	1.720795656
8	10.179587433	12.326668813	8.568364931
8	-0.784627451	3.965236178	5.140494321
8	-0.789447411	3.958305777	12.020724007
8	8.635893811	3.873735306	5.142414111
8	8.591437650	3.951153436	12.023760534
8	-5.481739669	12.116444524	5.142516048
8	-5.482811924	12.093832499	12.022913717
8	3.889189905	12.142634569	5.143263306
8	3.887014022	12.110307300	12.022324787

8	1.632950223	4.807258781	1.680558908
8	1.632698180	4.807898957	8.580283574
8	11.008855319	4.791277049	1.700753479
8	11.010750463	4.791561317	8.556120595
8	-3.080440403	12.942216103	1.688893661
8	-3.081406080	12.941483197	8.567236700
8	6.316347861	12.984523550	1.634376077
8	6.315184154	12.985362431	8.619520172
8	3.069307270	3.315362534	5.145878165
8	3.071481206	3.356045165	12.030647060
8	12.454973268	3.331230029	5.151854799
8	12.470798886	3.331163702	12.031738271
8	-1.657423460	11.479472007	5.150370885
8	-1.624610479	11.483065423	12.032320120
8	7.783375148	11.508006980	5.149523619
8	7.771525835	11.495334994	12.034110745
8	-0.265180485	7.158370337	1.669846863
8	-0.265034436	7.156864677	8.583052015
8	9.128217925	7.133408616	1.706235004
8	9.129278321	7.132654404	8.556116577
8	-4.970828402	15.289372333	1.676237483
8	-4.972097157	15.288486870	8.584108761
8	4.418601620	15.315482942	1.658338955
8	4.417674794	15.314833122	8.603056733
8	4.957925335	0.996844700	5.150500892
8	4.958689491	0.985481483	12.031024193
8	14.355455748	1.007059590	5.150151038
8	14.349321279	0.994860541	12.032024325
8	0.271865924	9.171615871	5.150602846
8	0.266913398	9.140083428	12.033635280
8	9.696144705	9.164544687	5.147128665
8	9.661490383	9.151797439	12.030150389
8	-1.345519470	4.347437335	1.690998855
8	-1.346440686	4.347789283	8.569712118
8	8.035960028	4.323402163	1.693979407
8	8.037429286	4.323722261	8.556743994
8	-6.044862964	12.485944500	1.686343948
8	-6.045480793	12.485797763	8.570057431
8	3.343147369	12.494917029	1.677737543
8	3.342645269	12.496764170	8.581738403
8	6.074619632	3.766188472	5.150059445
8	6.034825445	3.794556809	12.033956166
8	15.443711618	3.800534237	5.150691747
8	15.436289415	3.805927066	12.031050456
8	1.352709639	11.881109110	5.146475165
8	1.333072458	11.955893634	12.032080251
8	10.740348213	11.957164144	5.151291390
8	10.743758976	11.949959389	12.030827205
8	0.794036076	2.777833463	0.457598192
8	0.797156833	2.787464617	7.339626492

8	10.188255396	2.765746990	0.455048127
8	10.192410303	2.733230443	7.350452730
8	-3.908459832	10.911859730	0.455379543
8	-3.898219193	10.897183674	7.345644468
8	5.489670642	10.923294742	0.442695103
8	5.487512336	10.991887690	7.372476470
8	3.918076209	5.312045801	6.396927513
8	3.893654293	5.387527383	13.268764343
8	13.297739377	5.346855791	6.387767767
8	13.290050324	5.358263535	13.269260319
8	-0.789038080	13.478833025	6.389293272
8	-0.803947556	13.512321239	13.268043687
8	8.572382318	13.553155666	6.385971713
8	8.594677651	13.526409532	13.265195537
8	1.929036954	7.479896415	0.474427777
8	1.892284165	7.434246709	7.331418364
8	11.283075084	7.431458643	0.446933747
8	11.322362975	7.456264141	7.355177431
8	-2.791165960	15.592333402	0.457977804
8	-2.806969008	15.576434095	7.343094780
8	6.615939023	15.625433238	0.466709058
8	6.561882958	15.588036296	7.329705974
8	2.789510311	0.691485532	6.387716049
8	2.791778703	0.697293246	13.267959912
8	12.189894581	0.697966304	6.388609363
8	12.177798632	0.698833641	13.267346822
8	-1.889795766	8.846851338	6.387776248
8	-1.901614308	8.840960268	13.267468184
8	7.399032532	8.877693254	6.437822282
8	7.497891255	8.840375652	13.270310153
8	-2.698009881	6.073168057	0.460936826
8	-2.695633544	6.075371220	7.337241559
8	6.702996796	6.043915603	0.436367240
8	6.642208471	6.059495518	7.379053441
8	-7.412436710	14.211865640	0.467551045
8	-7.394893010	14.213484315	7.336510079
8	1.973752111	14.220446155	0.463199394
8	1.997421607	14.210703846	7.334899104
8	7.397974360	2.028431137	6.389865734
8	7.387789955	2.071909430	13.267090201
8	16.799477365	2.079289409	6.385644162
8	16.783033533	2.083018187	13.267720632
8	2.814385818	10.197314104	6.375153687
8	2.678063813	10.233957412	13.266624446
8	12.103131860	10.238810619	6.387744250
8	12.084659435	10.220130716	13.268081650
8	3.917349315	5.312193285	3.891693973
8	3.893763183	5.376957477	10.775407747
8	13.297733074	5.331177237	3.891968500
8	13.289773396	5.346299286	10.774846028

8	-0.790728107	13.467031938	3.891976253
8	-0.804411668	13.497684138	10.773755076
8	8.572511451	13.542289612	3.894888024
8	8.593692672	13.507745478	10.773591213
8	0.796402038	2.810675282	2.952652360
8	0.794522694	2.801129634	9.834707037
8	10.191321166	2.760318144	2.944512288
8	10.187976363	2.796381209	9.839558870
8	-3.898510652	10.928241766	2.950072825
8	-3.908018189	10.939843327	9.839463484
8	5.489317995	11.022770483	2.924000315
8	5.488483208	10.955339858	9.855319904
8	2.800528604	0.696183051	3.893404909
8	2.802401515	0.702438276	10.775119840
8	12.201239665	0.704735838	3.891634471
8	12.190053218	0.706177620	10.774405565
8	-1.877628516	8.852080928	3.892013389
8	-1.886747477	8.847572088	10.773826221
8	7.401429418	8.879173640	3.850068285
8	7.505276289	8.844789422	10.776857735
8	1.863513365	7.418172871	2.964684820
8	1.901994485	7.464369194	9.822833502
8	11.302010352	7.442732186	2.938306127
8	11.263330097	7.419882024	9.845862867
8	-2.827279114	15.565095385	2.950376941
8	-2.814419044	15.579045281	9.835830043
8	6.542837175	15.576358229	2.961349343
8	6.595981280	15.612199639	9.825902080
8	7.389975353	2.034461745	3.893535787
8	7.376276413	2.081874328	10.775647871
8	16.788713129	2.085606991	3.895584151
8	16.772128465	2.088619700	10.775132456
8	2.811219679	10.198597571	3.911270581
8	2.667628001	10.240209261	10.778050215
8	12.093149587	10.248016689	3.892165837
8	12.075583962	10.227287521	10.774768747
8	-2.672015531	6.062930728	2.955912172
8	-2.676723405	6.061709148	9.832151148
8	6.672921314	6.042266271	2.922465047
8	6.736009005	6.024036612	9.863713398
8	-7.369712726	14.199000307	2.957982039
8	-7.389068460	14.196738956	9.827276013
8	2.020757769	14.197622966	2.957021876
8	1.992599983	14.209467305	9.829179808
9	0.000258552	0.013472406	1.740171102
9	-0.000846347	0.013550218	8.600672388
9	9.383396870	-0.010093879	1.727026751
9	9.382014601	-0.009151215	8.616047179
9	-4.695429777	8.143217416	1.719295195
9	-4.695048482	8.143292487	8.622609754

9	4.691542480	8.173600453	1.762221144
9	4.692731110	8.175106380	8.590658008
9	-0.000796403	0.002794483	5.144012845
9	0.004373728	0.004679679	12.023106744
9	9.396634280	-0.041938025	5.144337329
9	9.387473005	0.000418346	12.023649966
9	-4.701352644	8.132627953	5.141547163
9	-4.697616427	8.139793174	12.024816677
9	4.632022295	7.989961681	5.152073704
9	4.694880289	8.153170041	12.026040503

S7.2. Ce2-F

58	5.814436402	6.415898569	5.154174447
9	4.717061900	8.177729329	3.915922656
9	4.572558485	8.136650384	6.340246459
20	9.451200888	5.409227420	6.854198335
20	4.690200336	2.710796914	13.734267468
20	4.715808707	2.704070023	6.877057813
20	14.098747399	2.693531233	13.739061175
20	14.102531182	2.700367066	6.870631225
20	0.006723352	10.839450294	13.735388757
20	0.012648384	10.833758935	6.860125775
20	9.394952301	10.845882649	13.739695375
20	9.409979557	10.893190364	6.874051353
20	-0.008455269	5.424005702	6.852616781
20	0.018228895	5.436077060	13.728706609
20	9.378137278	5.427364998	13.720324834
20	-4.705078804	13.547162693	6.845603353
20	-4.701602591	13.547400969	13.723042424
20	4.685329282	13.549196245	6.850673381
20	4.690392693	13.533074274	13.724378416
20	8.213652174	1.960614733	1.706987475
20	8.215355668	1.963255717	8.573431961
20	17.610498106	1.975229033	1.698932940
20	17.613142602	1.974166767	8.579064489
20	3.544434393	10.058807261	1.713574598
20	3.539885792	10.053989617	8.554644020
20	12.900816924	10.106679073	1.699742957
20	12.907461406	10.108443962	8.581980383
20	3.575328357	6.173175279	1.686578251
20	3.570507771	6.178701152	8.586982619
20	12.973096554	6.121706729	1.694366310
20	12.974209986	6.121694597	8.583050213
20	-1.118734975	14.250714695	1.695858321
20	-1.115611277	14.251409820	8.575746046
20	8.280578829	14.250208607	1.706023238
20	8.280367384	14.254376666	8.570360444
20	1.100009489	1.990396264	5.137175798
20	1.119725133	2.012898046	12.013237994
20	10.532720014	2.021154185	5.141496979

20	10.516796617	2.005543044	12.015689198
20	-3.559480627	10.151160158	5.136293154
20	-3.575751866	10.141962941	12.010352253
20	5.716921831	10.067393558	5.122074264
20	5.804881311	10.129070165	12.011400591
20	2.303620700	0.036494999	1.696720850
20	2.305964957	0.039495308	8.577413585
20	11.708414183	0.024255374	1.698598919
20	11.709574749	0.020546038	8.583062705
20	-2.402038615	8.176001296	1.703582383
20	-2.402302366	8.177037660	8.572631136
20	6.955018358	8.181973880	1.662479109
20	6.962861354	8.185070266	8.608258396
20	2.490159635	8.162454502	5.141772351
20	2.411292707	8.096194327	12.012335111
20	11.803118142	8.091988340	5.148368022
20	11.790063835	8.086740337	12.019306054
20	-2.317270514	16.240597990	5.138215986
20	-2.297206683	16.226074476	12.013574196
20	7.076155818	16.221348989	5.135804598
20	7.096997612	16.222639130	12.011461478
20	-3.500609701	6.164638331	5.138293683
20	-3.510855502	6.159007131	12.013494882
20	5.866537554	6.162086838	12.017184691
20	-8.228036005	14.272455400	5.133568790
20	-8.212920368	14.286540744	12.011045957
20	1.169659057	14.327599635	5.133714838
20	1.188009736	14.284984576	12.010816727
20	-0.012688435	5.414846515	3.426154958
20	0.010881450	5.429819630	10.299175730
20	9.459714294	5.406252357	3.408642631
20	9.391482348	5.431783318	10.300144875
20	-4.696178740	13.553883499	3.422862202
20	-4.688713651	13.554759368	10.297296796
20	4.686390405	13.549588382	3.422145410
20	4.697656409	13.527137822	10.295300091
20	4.715006883	2.691219773	3.405868153
20	4.691480988	2.701208852	10.299035288
20	14.103679105	2.711993528	3.413922632
20	14.098297118	2.710033641	10.294355725
20	-0.003475861	10.835130558	3.408792471
20	0.005513562	10.841848428	10.287779703
20	9.365267474	10.847623636	3.416547967
20	9.372972632	10.835021058	10.292722072
15	1.600414995	3.246682019	1.708571030
15	1.601175574	3.244178740	8.569106561
15	10.992554108	3.228595700	1.697638390
15	10.992100791	3.233810125	8.579239709
15	-3.109321097	11.373743266	1.696936929
15	-3.103582752	11.375315886	8.571178866

15	6.288365557	11.391629118	1.694881284
15	6.288003222	11.373188230	8.560021782
15	3.075960266	4.847322995	5.142207413
15	3.107009675	4.884099042	12.011777589
15	12.519644797	4.890789556	5.140833711
15	12.499859159	4.886964577	12.017038289
15	-1.596015637	13.047131728	5.137575158
15	-1.591310247	13.022702688	12.012388988
15	7.778536328	12.975454121	5.141661182
15	7.798668284	13.023123198	12.013365595
15	1.074498273	7.888755576	1.702563036
15	1.065353718	7.885411408	8.576749862
15	10.469414487	7.887810994	1.708996734
15	10.472707898	7.890017395	8.570982167
15	-3.604985553	16.024066588	1.694891559
15	-3.603293962	16.023740660	8.575582774
15	5.800257703	16.020017138	1.700207092
15	5.800485568	16.018476981	8.575852214
15	3.592570257	0.236302760	5.138408818
15	3.609611709	0.238512408	12.014796186
15	12.999227192	0.240624331	5.141050531
15	13.003853596	0.231727871	12.012321592
15	-1.076969129	8.373821450	5.136008613
15	-1.091957601	8.372524388	12.012284229
15	8.315109577	8.358179736	5.124827131
15	8.323527531	8.384708607	12.013899434
15	-2.689752439	5.137214783	1.696166200
15	-2.688993269	5.136235363	8.575963795
15	6.723146692	5.112727495	1.672340109
15	6.728187810	5.109906044	8.619568313
15	-7.387638470	13.266614179	1.700425192
15	-7.384568639	13.271994538	8.580828002
15	2.010720316	13.256887504	1.701774803
15	2.013209155	13.254083059	8.571144440
15	7.370917078	3.037322418	5.139326436
15	7.387479499	2.995793382	12.014500763
15	16.775426079	2.994162977	5.141532537
15	16.784988126	2.997166200	12.015509968
15	2.640193043	11.184938046	5.137542766
15	2.684719488	11.123117527	12.009472909
15	12.080924916	11.118479950	5.136125494
15	12.081059490	11.124452554	12.011165959
8	3.028795657	2.653909080	1.712026273
8	3.030194176	2.652058193	8.566946863
8	12.421933389	2.642700305	1.706253279
8	12.420972373	2.646349426	8.578669105
8	-1.678983648	10.789905494	1.694929959
8	-1.673828058	10.791159917	8.573740617
8	7.716688757	10.799857048	1.694577389
8	7.713524963	10.766354605	8.548067151

8	1.656731293	5.433690201	5.143282654
8	1.677069431	5.466245298	12.012070644
8	11.083562125	5.464784022	5.140803807
8	11.067830682	5.468156499	12.017571716
8	-3.036623735	13.612075487	5.140939887
8	-3.024209794	13.603742856	12.014715247
8	6.366445647	13.600142502	5.140817421
8	6.371158610	13.616294044	12.013321898
8	5.579976135	1.288639015	1.691615319
8	5.580267979	1.287771068	8.588347306
8	14.979958360	1.284328340	1.706797558
8	14.977839935	1.282086279	8.580544913
8	0.871084829	9.420129415	1.691559999
8	0.861708333	9.415838036	8.585883457
8	10.271071734	9.420474185	1.718376843
8	10.277699818	9.423159222	8.571534783
8	-0.896900872	6.838316920	5.135634002
8	-0.887922344	6.839615975	12.013024512
8	8.532622190	6.817073306	5.112314892
8	8.544744209	6.858090190	12.010346195
8	-5.582658487	14.973883383	5.134237228
8	-5.580974931	14.973876613	12.011342823
8	3.822406719	14.979702141	5.138436550
8	3.812106088	14.967235150	12.009081372
8	5.497875320	4.176244857	1.686127969
8	5.505739240	4.171676496	8.608378293
8	14.881196026	4.181165876	1.695775595
8	14.881686589	4.180486051	8.576352590
8	0.773445043	12.329876712	1.703603404
8	0.776398284	12.326584847	8.569484460
8	10.182416896	12.313631369	1.699614276
8	10.183542898	12.320352438	8.595536092
8	-0.792578943	3.943462556	5.140836468
8	-0.781397775	3.946303619	12.015552704
8	8.615619525	3.950584339	5.137559390
8	8.609392674	3.943510385	12.012791995
8	-5.491528600	12.073781301	5.130692603
8	-5.488578389	12.078132317	12.010209820
8	3.879390541	12.106691113	5.141548393
8	3.906792175	12.070417949	12.008525096
8	1.633758605	4.802394090	1.711838250
8	1.632797457	4.799916074	8.567657518
8	11.024310486	4.785791163	1.667731970
8	11.025030572	4.790709675	8.579631312
8	-3.083047757	12.930722811	1.695652205
8	-3.078551141	12.932198226	8.560076507
8	6.318341539	12.947034398	1.706408818
8	6.329028817	12.928405564	8.544602420
8	3.079575631	3.299360767	5.129878998
8	3.079288571	3.327227602	12.010303522

8	12.494042305	3.335286865	5.151413405
8	12.476548635	3.330184528	12.026818069
8	-1.610491160	11.489737375	5.139127656
8	-1.616928487	11.466541416	12.013102478
8	7.721694529	11.418555885	5.153586116
8	7.766156062	11.467460886	12.017663354
8	-0.286858796	7.134710125	1.737691561
8	-0.295180258	7.130374595	8.557182651
8	9.107182259	7.135914130	1.686650419
8	9.106457676	7.144393198	8.547646808
8	-4.965438235	15.268141907	1.693236076
8	-4.963380925	15.267408910	8.554393949
8	4.439796425	15.263199952	1.694779710
8	4.441318386	15.258787648	8.581082520
8	4.952199063	0.999533219	5.149317939
8	4.972935544	0.990276968	12.023342851
8	14.357114997	1.001833587	5.137168946
8	14.366826847	0.985553847	12.004987578
8	0.292128579	9.111401254	5.132632866
8	0.269536238	9.125298076	12.009787954
8	9.651358564	9.129289094	5.220204283
8	9.681050695	9.144636537	12.027847824
8	-1.353136269	4.338150202	1.690201874
8	-1.352697770	4.336464411	8.573724382
8	8.056442212	4.320465204	1.749062518
8	8.062316739	4.322461977	8.527944985
8	-6.051973830	12.467783122	1.708244940
8	-6.051301033	12.469357451	8.575964345
8	3.335380222	12.440951960	1.697461697
8	3.339179347	12.439972192	8.574072169
8	6.055097474	3.882675981	5.140847006
8	6.049835935	3.790745644	12.016988145
8	15.443142890	3.798386253	5.147376162
8	15.449217269	3.797239498	12.020248083
8	1.310661446	11.986953907	5.114342079
8	1.351862919	11.929329431	12.013173924
8	10.744525443	11.918811214	5.129997876
8	10.746078415	11.925462888	11.998541045
8	0.808463806	2.782984380	0.459707459
8	0.802791168	2.779923614	7.324266632
8	10.190473185	2.738382168	0.467682423
8	10.196644150	2.779298677	7.327884764
8	-3.907627627	10.907948843	0.452953978
8	-3.901207985	10.900949831	7.329552457
8	5.497195292	10.943343533	0.442481813
8	5.481429719	10.894670946	7.327092494
8	3.885251218	5.312403434	6.388733520
8	3.899527197	5.342784389	13.262218522
8	13.305297138	5.374835214	6.382571354
8	13.296840810	5.364474724	13.256680142

8	-0.806787715	13.530034382	6.378853973
8	-0.795919564	13.491371620	13.256073533
8	8.593319639	13.426564369	6.384762375
8	8.598502521	13.487362273	13.257624381
8	1.832581593	7.409489747	0.437681986
8	1.880963201	7.442233143	7.336913063
8	11.289381243	7.441448392	0.471946709
8	11.282267030	7.423926439	7.331967425
8	-2.797366638	15.568704668	0.454632795
8	-2.784733152	15.590251848	7.334537254
8	6.609402211	15.575225478	0.457703375
8	6.598131453	15.569029926	7.325803876
8	2.781501673	0.682326079	6.379597826
8	2.804244504	0.694906891	13.257190035
8	12.199407241	0.692398425	6.389064539
8	12.212866612	0.692663428	13.262727682
8	-1.872477550	8.840470901	6.382699633
8	-1.890244157	8.828317324	13.260311987
8	7.385173336	8.700666782	6.316522685
8	7.510087345	8.833694480	13.252156492
8	-2.704008793	6.065770496	0.457333988
8	-2.700699657	6.058022878	7.330843974
8	6.773916649	5.997256778	0.407211000
8	6.657216409	6.077565381	7.403205226
8	-7.388511125	14.184280503	0.451990757
8	-7.402804950	14.182075636	7.327194427
8	2.012456872	14.180080400	0.455087655
8	2.018973568	14.177892639	7.325009170
8	7.338773451	2.121466300	6.387797941
8	7.400817654	2.075684521	13.261879393
8	16.784870046	2.069172224	6.384810354
8	16.791139852	2.072100551	13.258847692
8	2.613840949	10.283172659	6.396412935
8	2.692008653	10.205243828	13.257796433
8	12.084483945	10.208519125	6.389548618
8	12.080488308	10.213615385	13.264705990
8	3.883550314	5.345207710	3.907620713
8	3.901895976	5.349992728	10.766027312
8	13.301834434	5.351037758	3.886703577
8	13.290657600	5.343989932	10.765269221
8	-0.815257311	13.528028877	3.890162969
8	-0.799669638	13.489941301	10.765269114
8	8.584658777	13.414200958	3.890172139
8	8.593539686	13.481133676	10.763770085
8	0.798925132	2.784569209	2.952652692
8	0.807706318	2.780022577	9.817188911
8	10.200109338	2.798774254	2.959601010
8	10.192317363	2.772182881	9.821541016
8	-3.901219424	10.910342594	2.945740804
8	-3.898340927	10.920644071	9.821513588

8	5.493839253	10.917957694	2.937758877
8	5.507211247	10.935925490	9.823264267
8	2.800479678	0.690167373	3.887660101
8	2.819367087	0.700123453	10.764410108
8	12.190187853	0.690086982	3.898243443
8	12.197168612	0.690459432	10.771284033
8	-1.875721162	8.843358057	3.891515915
8	-1.895471012	8.827297842	10.767117219
8	7.563090863	8.805161690	3.850583618
8	7.538349757	8.848122451	10.761234462
8	1.908750927	7.453365063	2.933923339
8	1.840572619	7.412517567	9.833427999
8	11.245188433	7.411544289	2.967071668
8	11.254167995	7.421642203	9.825258336
8	-2.809165879	15.580290186	2.947565450
8	-2.817563118	15.558694586	9.827157171
8	6.597928732	15.565677493	2.948997410
8	6.610924169	15.567533079	9.815805076
8	7.333554012	2.120231527	3.891034685
8	7.396091911	2.075935883	10.766601590
8	16.778050510	2.078142008	3.891762111
8	16.787644440	2.079718754	10.766817785
8	2.657797319	10.256370414	3.898054800
8	2.687467195	10.208006556	10.759233393
8	12.084515064	10.188608218	3.897532076
8	12.099958362	10.194163870	10.772636147
8	-2.697320768	6.049922311	2.948028979
8	-2.700013003	6.054850010	9.822199606
8	6.660957303	6.079637263	2.888308770
8	6.787612074	5.996227342	9.881566633
8	-7.404061228	14.189611082	2.944860600
8	-7.384272750	14.198676772	9.821148632
8	2.020836313	14.180355710	2.947571365
8	2.014991960	14.175923179	9.818297631
9	0.005326734	0.003624158	1.706849561
9	0.007349405	0.002962758	8.570603060
9	0.005366379	16.260964177	1.723493617
9	0.007805552	16.263542944	8.579382797
9	-4.707698747	8.136142386	1.713500819
9	-4.707256664	8.136587223	8.586399527
9	4.699234815	8.115118018	1.236649140
9	4.713857627	8.116374958	9.014136305
9	-0.017060158	-0.027802697	5.127483352
9	0.007189278	-0.002148753	12.005006864
9	9.374581238	0.043161978	5.139932666
9	9.401260469	-0.005039254	12.019225289
9	-4.686644391	8.139621359	5.138895345
9	-4.697393086	8.130810010	12.016479724
9	4.704154948	8.126920792	12.003893762

S7.3. Ce2_1-Vac

58	5.989536260	6.254715448	5.206259414
58	5.688379013	10.140998504	12.023013457
20	4.685868234	2.696900553	13.758309093
20	4.721371211	2.691190854	6.901275063
20	14.096137089	2.711741588	13.758466521
20	14.066941785	2.732999840	6.884717410
20	-0.066226426	10.864254237	13.765546357
20	-0.013068810	10.882571806	6.874496398
20	9.459407011	10.873520202	-0.005148291
20	9.499893785	10.889711187	6.894805850
20	-0.003897732	5.415586912	6.864400527
20	-0.000893346	5.423484998	13.750426305
20	9.429745417	5.398774294	13.732663648
20	-4.728910145	13.551785119	6.854181706
20	-4.703002886	13.561646689	13.747667667
20	4.685051987	13.616708915	6.849262182
20	4.680102772	13.654766753	13.764085271
20	8.224309643	1.976172686	1.712739781
20	8.259398674	1.987407644	8.577495773
20	17.626924699	1.981939885	1.706239984
20	17.624308154	1.980757281	8.585482145
20	3.501122423	10.129821348	1.744566218
20	3.497188653	10.137491674	8.539352647
20	12.905963651	10.096091906	1.718631137
20	12.892821815	10.050777753	8.579872892
20	3.587493575	6.135719278	1.695605819
20	3.613740535	6.133720942	8.628760862
20	12.988353063	6.122381338	1.710848794
20	12.938712330	6.128105674	8.560076050
20	-1.134135223	14.270442076	1.700751830
20	-1.146156449	14.255469367	8.586438787
20	8.286421653	14.280672190	1.708262023
20	8.285204375	14.284210376	8.580787970
20	1.108065843	2.018031431	5.145718285
20	5.910630187	6.051507560	12.045216828
20	1.106594637	2.009779128	12.033675918
20	10.513201163	2.081257583	5.201665888
20	10.518482515	2.014493711	12.031768432
20	-3.612221072	10.134664475	5.143326719
20	-3.598104021	10.145795661	12.024010444
20	5.815470597	10.236435113	5.133417907
20	2.303358236	0.035987962	1.702623235
20	2.306492847	0.040034258	8.591353254
20	11.696414710	0.063251755	1.719068418
20	11.667511867	0.097542119	8.577380971
20	-2.398030877	8.171764582	1.694266058
20	-2.387182396	8.164538919	8.591265583
20	7.051913768	8.159610143	1.693439692
20	7.048331502	8.119895508	8.573675887

20	2.309823471	8.132968884	5.144786624
20	2.346350807	8.052341772	12.028512248
20	11.732478277	8.051194956	5.198800690
20	11.792487596	8.091702131	12.032788195
20	-2.324923560	16.227109359	5.145139836
20	-2.297926006	16.240182636	12.027896515
20	7.100091487	16.260113210	5.142297269
20	7.089554499	16.269182137	12.029198329
20	-3.499047301	6.162530328	5.144409516
20	-3.531314194	6.154810297	12.025260525
20	-8.219017439	14.326110889	5.142077648
20	-8.214535980	14.315729099	12.031842931
20	1.168377596	14.348742874	5.143829044
20	1.186727156	14.298543915	12.022413958
20	-0.018090613	5.425170362	3.421450123
20	0.000176902	5.418498465	10.311765374
20	9.497532230	5.375916191	3.542142120
20	9.454024191	5.400039231	10.150077615
20	-4.706804440	13.568372508	3.424548665
20	-4.706654943	13.565904902	10.308868349
20	4.689892338	13.591302636	3.430351304
20	4.686442821	13.655962915	10.288613470
20	4.732159849	2.644877658	3.417637779
20	4.677870773	2.660777063	10.316476112
20	14.102681976	2.767757488	3.428663381
20	14.116217591	2.736315198	10.305517241
20	-0.013141360	10.884685835	3.416825603
20	-0.069455451	10.866583090	10.293539195
20	9.400064188	10.857488428	3.428582394
20	9.429082787	10.882336602	10.298064029
15	1.604097338	3.252564792	1.709215013
15	1.599236090	3.243824172	8.585974515
15	11.008498652	3.238260555	1.698087720
15	11.035293864	3.188175689	8.620149135
15	-3.124653896	11.373891179	1.705952792
15	-3.135674463	11.371215976	8.576319716
15	6.308211007	11.429254564	1.722417537
15	6.314048851	11.404962947	8.534155134
15	3.104007815	4.911144766	5.142693869
15	3.097676502	4.883694529	12.052250384
15	12.567429438	4.929650857	5.115391370
15	12.515692589	4.893017358	12.025867912
15	-1.608170822	13.040543036	5.143523160
15	-1.600397118	13.031006634	12.032179589
15	7.809805842	13.042654876	5.156607212
15	7.797537393	13.030329655	12.036229347
15	1.103351040	7.918568441	1.693849287
15	1.106624482	7.909689950	8.593644863
15	10.491646055	7.893103993	1.694035659
15	10.522775085	7.952513455	8.628231302

15	-3.622993277	16.018514926	1.684068377
15	-3.619562588	16.021262493	8.588484138
15	5.797608381	16.058532498	1.705336614
15	5.800339335	16.058344734	8.582750943
15	3.582455246	0.229922058	5.153031833
15	3.598950817	0.223945339	12.042223003
15	13.017317871	0.258373834	5.132840529
15	13.005594680	0.249336434	12.041755244
15	-1.115712190	8.389494283	5.142438302
15	-1.071226475	8.397648760	12.031165669
15	8.259267906	8.392907206	5.083141948
15	8.297729299	8.364002532	12.024043096
15	-2.663007851	5.137507769	1.684127121
15	-2.666441559	5.136344937	8.590851388
15	6.719195317	5.111663119	1.677562441
15	6.654272895	5.095958960	8.682830426
15	-7.392200669	13.284064117	1.702349194
15	-7.391271841	13.293868263	8.594484775
15	2.012666784	13.287800464	1.693150087
15	2.018469055	13.293531416	8.589447168
15	7.387838471	2.952113185	5.112244623
15	7.398760460	2.969739090	12.029235613
15	16.822335554	2.991956177	5.150969119
15	16.809272557	2.997767729	12.038370665
15	2.657836261	11.175056348	5.143183864
15	2.719294832	11.129287080	12.030517747
15	12.072918494	11.152987544	5.128308806
15	12.095901133	11.146019367	12.044056333
8	3.032754187	2.662135257	1.713377382
8	3.012966402	2.617732725	8.582739831
8	12.441513526	2.654181640	1.691206589
8	12.466834838	2.559391621	8.646316502
8	-1.688773759	10.793695562	1.715015581
8	-1.695166494	10.798164729	8.570962020
8	7.730809724	10.843370568	1.693604255
8	7.729264466	10.788028848	8.538683769
8	1.680580053	5.503031315	5.134518670
8	1.672958544	5.487972546	12.056637608
8	11.212853603	5.619160419	5.038005472
8	11.082186580	5.465582906	12.014246771
8	-3.035007991	13.637588863	5.144773416
8	-3.022049032	13.629857411	12.030494778
8	6.367691727	13.604300582	5.145388241
8	6.348393660	13.562601168	12.032641075
8	5.559089765	1.260001782	1.671728921
8	5.574268209	1.266914867	8.600513083
8	15.014311580	1.303962200	1.705267321
8	15.055241047	1.307727820	8.591109126
8	0.895723917	9.453010829	1.678410068
8	0.892637583	9.444537916	8.612493772

8	10.241643427	9.423995158	1.685565989
8	10.343707070	9.507899909	8.656136409
8	-0.852650783	6.865730364	5.143299893
8	-0.839813305	6.871563370	12.030237849
8	8.450328751	6.879300707	4.954020115
8	8.509694299	6.846915069	12.019498137
8	-5.617741174	14.982575917	5.139844192
8	-5.609184312	14.976040054	12.035525083
8	3.827436986	15.007506061	5.124978019
8	3.830787784	15.002684516	12.043544389
8	5.500103990	4.163590368	1.693787577
8	5.382578169	4.198449260	8.660135124
8	14.926104537	4.188950272	1.673782061
8	14.914317770	4.196547523	8.588464157
8	0.793222095	12.335609966	1.686433305
8	0.802185796	12.334708108	8.598340165
8	10.189261589	12.337523809	1.707012189
8	10.160613772	12.385546090	8.600413173
8	-0.764782191	3.950569597	5.142094458
8	-0.774722500	3.947920350	12.037867057
8	8.629914544	3.824260932	5.029421010
8	8.584406713	3.956208859	12.019805272
8	-5.490514675	12.076040624	5.122556634
8	-5.479559328	12.082832962	12.050281984
8	3.891925946	12.104810109	5.145425863
8	3.907300157	12.134651114	12.029644126
8	1.634118542	4.811628528	1.717068975
8	1.654970487	4.801183800	8.584750778
8	11.041801074	4.793667578	1.722505240
8	11.130732399	4.717910834	8.605347525
8	-3.090809029	12.929887730	1.701138860
8	-3.107024007	12.927986059	8.563882403
8	6.315270453	12.978380999	1.679748193
8	6.338619000	12.955218173	8.554368768
8	3.091588285	3.360671507	5.142047286
8	3.058205585	3.328561953	12.021817369
8	12.472272129	3.370086262	5.168240921
8	12.500850488	3.334247683	12.036648172
8	-1.645201469	11.484054841	5.147336677
8	-1.645386115	11.473802680	12.032394886
8	7.799964850	11.485259129	5.207337624
8	7.784547576	11.460421534	12.043374947
8	-0.254100885	7.161648139	1.696867173
8	-0.248937922	7.148980774	8.574457700
8	9.144439184	7.114657766	1.719540588
8	9.150027429	7.265344426	8.631148661
8	-4.985208201	15.263126326	1.685935057
8	-4.985422371	15.271570761	8.561788501
8	4.427309653	15.315523970	1.731385500
8	4.418423893	15.338959039	8.564367065

8	4.946016058	0.975739665	5.189791834
8	4.965089899	0.966964034	12.053790558
8	14.380516283	1.013406805	5.138794808
8	14.367541740	1.005214281	12.012141347
8	0.230058218	9.178671052	5.146594153
8	0.287218796	9.169508310	12.030264716
8	9.599580104	9.155051673	5.232058668
8	9.635632379	9.148165356	12.031890259
8	-1.327250407	4.335031614	1.679902952
8	-1.333614435	4.326861711	8.582837369
8	8.056260441	4.335218354	1.730838717
8	7.928241412	4.257657851	8.698672133
8	-6.061583077	12.476937273	1.704345570
8	-6.082178900	12.455016402	8.594247271
8	3.350537839	12.487069575	1.695159066
8	3.359597908	12.495299365	8.573967363
8	6.060754095	3.805448232	5.168484328
8	6.034957485	3.734164092	12.026249951
8	15.491463915	3.789250950	5.184642342
8	15.476379675	3.797699237	12.043979003
8	1.331467460	11.984300784	5.147038666
8	1.364903480	11.873209492	12.026715086
8	10.752103546	11.981264372	5.131496910
8	10.765723094	11.962106256	12.013157012
8	0.813426058	2.793962352	0.457931418
8	0.797441952	2.798043437	7.337534774
8	10.209787259	2.800884503	0.442595677
8	10.266764068	2.554679048	7.438628386
8	-3.908917220	10.902318053	0.456982328
8	-3.930816223	10.892950843	7.337643846
8	5.499309460	10.932334644	0.486696493
8	5.483749016	10.938430234	7.318598318
8	3.901244499	5.394073089	6.389808528
8	3.895409797	5.306838761	13.312708405
8	13.378701371	5.384587490	6.360110550
8	13.303296943	5.366499543	13.279215928
8	-0.806991629	13.503763957	6.387592418
8	-0.792248569	13.473632216	13.279000211
8	8.606595787	13.557127287	6.380223988
8	8.587360288	13.495839843	13.279487416
8	1.912234731	7.454614692	0.455362629
8	1.926454208	7.478641824	7.351984621
8	11.275473126	7.441273804	0.435332678
8	11.440326612	7.612013919	7.436172114
8	-2.818453863	15.553178257	0.444271520
8	-2.804240490	15.573536461	7.350902458
8	6.561299090	15.577713548	0.440642379
8	6.601675212	15.580417433	7.344285402
8	2.754429591	0.679602246	6.381861640
8	2.790071231	0.684441968	13.281583484

8	12.222486908	0.693852797	6.383461957
8	12.235994050	0.703315233	13.305345655
8	-1.927693254	8.823698218	6.385564869
8	-1.866748922	8.853948923	13.279591032
8	7.302443365	8.678894820	6.279340043
8	7.459368143	8.824254409	13.263646696
8	-2.661540523	6.066028962	0.441486878
8	-2.654109969	6.061265214	7.347619714
8	6.726917944	6.031931938	0.433910697
8	6.550436872	6.058477058	7.462657977
8	-7.404215773	14.198786094	0.451611175
8	-7.386133807	14.205908132	7.342398463
8	2.020409164	14.203394815	0.442351111
8	1.996818765	14.215904596	7.346006135
8	7.375968733	2.036123815	6.362785184
8	7.417807542	2.063931691	13.290234614
8	16.869175302	2.050097977	6.378648719
8	16.823104312	2.068938322	13.279316958
8	2.651196572	10.246391469	6.384075705
8	2.839441141	10.193651461	13.261045075
8	12.053917128	10.254847632	6.387121673
8	12.053493908	10.247851383	13.307404660
8	3.925158974	5.404733237	3.915680085
8	3.890365490	5.380207693	10.822531033
8	13.437332459	5.262680838	3.859133702
8	13.329449687	5.356175667	10.788790680
8	-0.811553544	13.496679487	3.892973705
8	-0.793656407	13.472658834	10.783294922
8	8.587398950	13.475361264	3.888245320
8	8.582659837	13.483948608	10.784933095
8	0.804000275	2.787126728	2.951800896
8	0.798968247	2.796216357	9.835966715
8	10.217465703	2.736360083	2.930141395
8	10.217279866	2.782375545	9.885308850
8	-3.920170148	10.911116108	2.950925244
8	-3.911931659	10.918091499	9.835635387
8	5.499998190	10.984138237	2.962485651
8	5.519973625	10.955312612	9.798364514
8	2.816728738	0.704049546	3.891122176
8	2.811538787	0.691771263	10.790694262
8	12.214892143	0.711341474	3.891880985
8	12.172712197	0.666627358	10.812168195
8	-1.920147603	8.824422527	3.893958007
8	-1.868209502	8.854041118	10.783124560
8	7.516520699	8.960145381	3.833764991
8	7.454729031	8.843549901	10.798287732
8	1.909946403	7.478921563	2.941948287
8	1.894948171	7.440678948	9.842964359
8	11.338353825	7.491681092	2.922144398
8	11.293259986	7.449179666	9.886155295

8	-2.831765462	15.568454435	2.938068488
8	-2.852339800	15.543050234	9.847513556
8	6.615282100	15.591756441	2.937252663
8	6.561902183	15.558223869	9.843509978
8	7.238042639	2.034210483	3.861416804
8	7.416974883	2.025891233	10.801249743
8	16.820403060	2.087527537	3.892915121
8	16.820504987	2.076054529	10.792313599
8	2.649062745	10.255994396	3.894962711
8	2.850904274	10.190052569	10.802754106
8	12.039925092	10.229827988	3.887321996
8	12.118738331	10.210788948	10.814398576
8	-2.649231118	6.048437108	2.938668964
8	-2.642106816	6.043886643	9.849029451
8	6.643512990	6.074514003	2.897587491
8	6.667533163	6.061899783	9.898085328
8	-7.402848860	14.211296681	2.943418877
8	-7.379126756	14.215689948	9.840293304
8	2.000095127	14.204942428	2.940909097
8	2.036200974	14.194442561	9.848707139
9	-0.000011857	0.001902136	1.694717810
9	0.002063168	0.002495930	8.603674301
9	-0.012173317	16.272137989	1.695845961
9	-0.028390903	16.223892442	8.655567466
9	-4.688395162	8.134664878	1.690099630
9	-4.651218024	8.129721239	8.654958633
9	4.728988574	8.170918375	1.693018430
9	4.703825115	8.191146189	8.637863490
9	0.000341885	0.000783023	5.117953698
9	-9.412927097	16.272799305	12.027150369
9	9.383385514	0.021858368	5.129130564
9	9.411507327	-0.036658688	12.064675967
9	14.067561984	8.131332650	5.125378646
9	-4.697079347	8.143448445	12.059836485
9	4.615077306	8.215641611	5.096614825
9	4.675508248	8.015858171	12.065755263

S7.4. Ce2_2-Vac

58	5.964974512	6.355300232	5.268592441
58	5.724540897	10.098065736	11.943750879
20	-0.004112281	10.869108859	6.871073472
20	5.802595846	10.253355547	5.154746800
20	9.393769614	5.447266560	6.903526884
20	4.660820634	2.695333296	13.766574551
20	4.724414973	2.678091658	6.903257384
20	14.095019434	2.715372756	13.756987430
20	14.119326426	2.716017686	6.873832320
20	-0.023646805	10.839306770	13.743873710
20	9.448718523	10.883939333	-0.017701991
20	9.452569888	10.902078850	6.917793390

20	-0.015813305	5.469137331	6.873495774
20	0.009485331	5.417747826	13.752699192
20	9.422567168	5.375904628	13.728211647
20	-4.731061016	13.561922815	6.850558487
20	-4.713730649	13.574620250	13.742368904
20	4.651399236	13.592629942	6.846416558
20	4.683546117	13.639486962	13.754232134
20	8.227289137	1.961696612	1.713167220
20	8.238290775	1.921121858	8.585146215
20	17.632279300	1.974042826	1.711198358
20	17.625965595	1.962790113	8.590538344
20	3.510685248	10.123957901	1.745108365
20	3.558605670	10.129162857	8.537201049
20	12.901575017	10.116694414	1.702578895
20	12.890532064	10.064804430	8.590408020
20	3.584879158	6.139364496	1.693191303
20	3.573496658	6.125477363	8.648890344
20	12.996258036	6.134207500	1.707292905
20	12.971896208	6.100188515	8.596781631
20	-1.137245722	14.268398541	1.694383988
20	-1.146289336	14.243845956	8.575525396
20	8.275714475	14.275924446	1.701708426
20	8.261623806	14.270917794	8.584892462
20	1.137226354	2.029043041	5.152569378
20	1.109721491	1.992709448	12.032887036
20	10.539300985	2.011640681	5.149699346
20	10.512750089	1.967572227	12.027576046
20	-3.614685360	10.160986583	5.134370058
20	-3.605942034	10.142847861	12.020837622
20	2.300895503	0.031200503	1.712017111
20	2.302894940	0.027692354	8.600032816
20	11.706808857	0.029331831	1.703722068
20	11.693651991	0.053056215	8.589348753
20	-2.405059587	8.191062193	1.693553014
20	-2.433990543	8.185326369	8.584956747
20	7.043502057	8.156523386	1.660667728
20	2.290799994	8.127937512	5.156314215
20	2.352193012	8.038435449	12.016086786
20	11.769792961	8.100384302	5.170264733
20	11.819548319	8.086721461	12.034276377
20	-2.318635810	16.254628437	5.141578344
20	-2.307961860	16.221734659	12.019696724
20	7.102909363	16.269971685	5.144756167
20	7.096216028	16.263733174	12.016865697
20	-3.539667515	6.182422090	5.147912345
20	-3.524021215	6.140909672	12.024153060
20	5.938436775	6.042714203	12.013917871
20	-8.233237895	14.330081748	5.144575114
20	-8.224809384	14.305058538	12.014450888
20	1.159759117	14.334143420	5.135477443

20	1.188107888	14.263138997	12.008433748
20	-0.023102427	5.432583936	3.433698394
20	0.006613967	5.438821431	10.311266437
20	9.481699016	5.389181322	3.410710472
20	9.330204651	5.475251580	10.299629276
20	-4.712783823	13.581049435	3.419804795
20	-4.724160431	13.581042218	10.311630182
20	4.687312633	13.599099110	3.427682822
20	4.696366858	13.642218601	10.284344218
20	4.717480737	2.670066202	3.428417850
20	4.724530668	2.681634539	10.310427988
20	14.122969777	2.708123235	3.420276138
20	14.083288810	2.722963025	10.304932097
20	-0.015613636	10.878542096	3.407849050
20	-0.104174092	10.864973034	10.273058377
20	9.391913530	10.825785405	3.425785255
20	9.349395523	10.741222975	10.258283683
15	1.596328102	3.258777972	1.721894380
15	1.592195464	3.224158191	8.590960289
15	11.009975063	3.228132555	1.703156311
15	11.005969609	3.191281747	8.587691750
15	-3.126316714	11.390690663	1.695296902
15	-3.142420340	11.373748024	8.564881587
15	6.304892973	11.422884304	1.694380489
15	6.321954995	11.493102744	8.516480147
15	3.111928113	4.943428494	5.155477249
15	3.111100451	4.850941571	12.049661746
15	12.520575304	4.875220679	5.152241560
15	12.500291278	4.881627345	12.033903912
15	-1.614820931	13.041918559	5.133984699
15	-1.604264845	13.020481834	12.027401742
15	7.801001101	13.059609223	5.145094785
15	7.802119076	13.052840438	12.028550741
15	1.087215192	7.907493299	1.697009287
15	1.048904620	7.895282183	8.604239868
15	10.492110174	7.882089281	1.694531641
15	10.528253717	7.873992486	8.586595874
15	-3.618238153	16.046082251	1.689618776
15	-3.621636565	16.020996850	8.578717860
15	5.794362832	16.068234971	1.695209064
15	5.782739223	16.078640551	8.580183705
15	3.614400985	0.239951244	5.152081000
15	3.614176005	0.224872845	12.047936591
15	13.007265128	0.233204536	5.138149654
15	13.026923123	0.244506637	12.034697729
15	-1.132486054	8.399749147	5.135071641
15	-1.076546016	8.394762639	12.023027719
15	8.311883907	8.374698288	5.041873703
15	8.320445664	8.383046253	12.053401174
15	-2.697817364	5.157150802	1.700130442

15	-2.690917620	5.145983078	8.586376880
15	6.709312614	5.118518553	1.712315754
15	6.785803158	5.033583509	8.649473050
15	-7.400206147	13.295803542	1.705694313
15	-7.381608376	13.287556679	8.600304124
15	2.011927606	13.278396991	1.689969124
15	1.990954544	13.297290029	8.582111225
15	7.378733432	3.011782669	5.150604730
15	7.420736480	2.936241447	12.042735907
15	16.804541320	3.007401042	5.150939138
15	16.814481312	2.987002442	12.036097126
15	2.648437565	11.167061662	5.129467500
15	2.731382154	11.117176238	12.034666492
15	12.074207333	11.145590938	5.133956572
15	12.088417853	11.137253289	12.023778128
8	3.024377209	2.667094674	1.729373872
8	3.028065344	2.645230924	8.598421201
8	12.433823704	2.629573950	1.717134806
8	12.452322178	2.630605365	8.570230562
8	-1.690955227	10.813407183	1.700882361
8	-1.704754677	10.794073549	8.545986185
8	7.742957787	10.884804041	1.658136140
8	7.778243878	11.036592549	8.521617762
8	1.677869258	5.509375926	5.145217813
8	1.691161742	5.464762015	12.050866635
8	11.085619392	5.443969168	5.169439361
8	11.081874843	5.496277254	12.025560626
8	-3.045005437	13.630501526	5.131549517
8	-3.023159732	13.628579234	12.031858325
8	6.361120586	13.631357521	5.136729685
8	6.356480364	13.597896209	12.044445326
8	5.599409886	1.280081595	1.687537426
8	5.597900607	1.252711874	8.598724771
8	15.016097187	1.301223924	1.692838621
8	15.013737014	1.312924569	8.610907781
8	0.869393184	9.437448627	1.681266937
8	0.906554115	9.438901116	8.633472707
8	10.261100096	9.410309584	1.694802550
8	10.339430019	9.419204400	8.577638079
8	-0.883266819	6.873514873	5.131503475
8	-0.862411877	6.864788938	12.033980712
8	8.594011354	6.841283817	5.015775172
8	8.491993127	6.850955442	12.081045498
8	-5.609505624	15.007604299	5.134180343
8	-5.602701000	14.991934178	12.046152193
8	3.790119673	14.998698843	5.122309686
8	3.824660133	15.012964068	12.020529005
8	5.490765910	4.172804757	1.746161402
8	5.526282581	4.116917256	8.600661585
8	14.889821010	4.198671486	1.703806794

8	14.899648635	4.185940658	8.590978240
8	0.793320340	12.327521003	1.676207178
8	0.762857426	12.353506329	8.582620433
8	10.203272330	12.320617543	1.720528240
8	10.202726412	12.342166729	8.628448309
8	-0.781229259	3.965349579	5.155903901
8	-0.792635881	3.971377842	12.030836288
8	8.588073534	3.969156730	5.147542069
8	8.620172006	3.912929322	12.027290425
8	-5.509874021	12.099734433	5.108141126
8	-5.505982955	12.107807612	12.028270625
8	3.886027929	12.093828943	5.115428362
8	3.902986134	12.137457052	12.055680701
8	1.627952004	4.815791766	1.744877260
8	1.610980082	4.781044848	8.577012617
8	11.052977745	4.784076188	1.672659334
8	11.019469889	4.744130771	8.629074424
8	-3.097900103	12.947918703	1.686765387
8	-3.110832991	12.929571202	8.580968573
8	6.282129388	12.976564415	1.703270172
8	6.259377847	13.054308696	8.537016064
8	3.107795527	3.391428317	5.162362692
8	3.065713394	3.297623860	12.000202340
8	12.507128592	3.317417330	5.127846999
8	12.451131352	3.325202575	12.043472793
8	-1.642922675	11.486011724	5.148544784
8	-1.653462457	11.464744338	12.032372986
8	7.786079477	11.500788372	5.180669446
8	7.784830997	11.488020218	11.990941903
8	-0.270000996	7.146411232	1.711612645
8	-0.334523048	7.180899675	8.595254326
8	9.150325058	7.094275826	1.621270962
8	9.214697102	7.083477519	8.588232628
8	-4.984918550	15.301207547	1.704509019
8	-4.988688325	15.278719145	8.571748131
8	4.424914258	15.324092226	1.730631359
8	4.411165090	15.339969832	8.540951392
8	4.982272139	0.985179709	5.164738291
8	4.980962605	0.968900417	12.033292288
8	14.373760229	0.980257719	5.139295375
8	14.384867046	1.005802125	12.038725768
8	0.219088681	9.176711330	5.146674246
8	0.287604680	9.153393261	11.989826949
8	9.613900056	9.150407630	5.357576521
8	9.693934360	9.109886176	12.085025511
8	-1.364205962	4.351322424	1.699320179
8	-1.354351048	4.344626164	8.592095268
8	8.045701631	4.327556420	1.704454378
8	8.117700539	4.217697571	8.610208632
8	-6.059000577	12.503997715	1.705747257

8	-6.056733267	12.469263698	8.591667007
8	3.352661587	12.486947011	1.721943500
8	3.322328949	12.494568892	8.599952286
8	6.038417128	3.821941773	5.156786651
8	6.068621687	3.709637867	12.093102410
8	15.471808653	3.810964692	5.139131569
8	15.469521253	3.772895592	12.034758446
8	1.325068897	11.983310500	5.151628465
8	1.372045984	11.849239329	11.909945228
8	10.747033044	11.958505817	5.162203629
8	10.746994114	11.925677793	11.958607809
8	0.811173425	2.817683834	0.460419920
8	0.803271347	2.740956654	7.347492708
8	10.208336361	2.747672392	0.469014228
8	10.226209315	2.733115163	7.327762849
8	-3.917001746	10.914454062	0.450329774
8	-3.940991952	10.920800910	7.320068701
8	5.504043412	10.946101958	0.444620423
8	5.497878608	11.027667772	7.295231097
8	3.903497559	5.442670031	6.404823675
8	3.902611685	5.248757460	13.320636041
8	13.322620656	5.319912081	6.400872683
8	13.306382745	5.335570077	13.280005920
8	-0.818738782	13.521923148	6.373762051
8	-0.783533678	13.464683643	13.266791969
8	8.596222040	13.569797640	6.373292616
8	8.596727476	13.465975934	13.290744010
8	1.882653667	7.438746864	0.448912218
8	1.802101666	7.421597983	7.338234011
8	11.366273099	7.460867759	0.486967285
8	11.367888748	7.436369185	7.345444074
8	-2.827609702	15.569042436	0.445321243
8	-2.822097304	15.571886779	7.329967699
8	6.557683511	15.584398933	0.435025142
8	6.614728474	15.647991253	7.348000895
8	2.811732563	0.699504397	6.394865763
8	2.830859144	0.699554450	13.300536233
8	12.203585249	0.680401112	6.382563543
8	12.226149399	0.686147396	13.285532733
8	-1.948315459	8.831513082	6.375859375
8	-1.843745039	8.866366385	13.283820637
8	7.166770374	8.591191173	6.046474101
8	7.496255815	8.862644030	13.296225173
8	-2.702912038	6.079874100	0.454089308
8	-2.688275449	6.060319744	7.334168925
8	6.674358353	6.042174627	0.473567559
8	6.819849121	5.885151639	7.362121192
8	-7.420222083	14.207727078	0.452289771
8	-7.389629964	14.200022974	7.344165631
8	2.035312194	14.179826623	0.426434022

8	1.996788370	14.207427423	7.327883515
8	7.377043358	2.080665529	6.387569031
8	7.487730417	1.997962532	13.272752813
8	16.797309657	2.085938254	6.396025227
8	16.838493043	2.066917914	13.282304881
8	2.654990514	10.242830675	6.373850999
8	2.727149028	10.242780690	13.312854070
8	12.109688660	10.223188689	6.375155488
8	12.055618576	10.261147044	13.303573820
8	3.926037345	5.444303909	3.930715791
8	3.919386196	5.341355981	10.822251984
8	13.301998424	5.364053389	3.904945921
8	13.315754188	5.317308200	10.788930538
8	-0.820040110	13.492568908	3.880241151
8	-0.806833072	13.456638963	10.771528187
8	8.586368518	13.504363640	3.886066712
8	8.578931033	13.574458205	10.801770643
8	0.792055716	2.781152115	2.957535543
8	0.799170007	2.763542696	9.839590690
8	10.205657708	2.805510790	2.961593070
8	10.235600474	2.640462089	9.812244047
8	-3.921998924	10.933813350	2.943183481
8	-3.909587801	10.885568758	9.816759047
8	5.521041273	10.916033291	2.929533514
8	5.535985360	11.038720650	9.783108668
8	2.836045338	0.721702155	3.899981399
8	2.800728371	0.667351165	10.805692533
8	12.205607800	0.688017443	3.892664106
8	12.227936157	0.692224047	10.788373334
8	-1.923951997	8.849678125	3.883666068
8	-1.895958011	8.848002518	10.787768916
8	7.800455980	8.904470506	3.674935736
8	7.533622371	8.891006355	10.827980045
8	1.903791948	7.474809971	2.939696879
8	1.799897962	7.373863555	9.857815574
8	11.233257315	7.421018638	2.978736184
8	11.372018519	7.469251853	9.834298274
8	-2.812633533	15.602050844	2.937966854
8	-2.830131455	15.550529409	9.826323547
8	6.617771033	15.622848472	2.929581616
8	6.538401300	15.591269346	9.845361016
8	7.360389442	2.102411931	3.895313393
8	7.410389117	2.029049290	10.787388993
8	16.813759103	2.085900416	3.904869940
8	16.841615452	2.060429769	10.795796351
8	2.612199125	10.257691888	3.876475267
8	2.979784821	10.125155463	10.863678443
8	12.030988403	10.226943428	3.883399785
8	12.159024294	10.174074456	10.816399446
8	-2.699179957	6.074967611	2.948785593

8	-2.704596881	6.067433915	9.832870050
8	6.710841279	6.058394801	2.957458486
8	6.810549321	5.846090504	9.942680451
8	-7.411660246	14.227051171	2.945292235
8	-7.363206752	14.218700460	9.839743617
8	1.973113705	14.215341420	2.922888766
8	1.986091834	14.226831002	9.823123722
9	-0.000397206	0.007842261	1.684386686
9	0.000039504	-0.010639882	8.616511643
9	9.406278473	-0.018820916	1.707798424
9	9.395085602	-0.067017808	8.559216753
9	-4.707076011	8.163982918	1.721313493
9	-4.702387589	8.128600150	8.577650723
9	4.725316251	8.155008364	1.700998379
9	4.401258871	8.134197109	8.600811748
9	0.018585673	0.024170552	5.123591178
9	0.007063172	-0.020022824	12.068039277
9	9.394299724	0.005563081	5.125898641
9	9.428581226	-0.078659168	12.024020187
9	14.073107779	8.136001280	5.125907953
9	-4.687027966	8.124399086	12.073415174
9	4.565010559	8.270519066	4.997493932
9	4.679206676	7.978255643	12.148598789
